# Identification of Chondrocyte Genes and Signaling Pathways in Response to Acute Joint Inflammation

**DOI:** 10.1038/s41598-018-36500-2

**Published:** 2019-01-14

**Authors:** Mengxi Lv, Yilu Zhou, Shawn W. Polson, Leo Q. Wan, Meiqing Wang, Lin Han, Liyun Wang, X. Lucas Lu

**Affiliations:** 1 Department of Mechanical Engineering, University of Delaware, Newark, DE United States; 2 Center for Bioinformatics and Computational Biology, University of Delaware, Newark, DE United States; 3 Department of Biomedical Engineering, Rensselaer Polytechnic Institute, Troy, NY United States; 4 Department of Oral Anatomy and Physiology and TMD, the Fourth Military Medical University, Xi’an, Shanxi China; 5 School of Biomedical Engineering, Science, and Health Systems, Drexel University, Philadelphia, PA United States

## Abstract

Traumatic joint injuries often result in elevated proinflammatory cytokine (such as IL-1β) levels in the joint cavity, which can increase the catabolic activities of chondrocytes and damage cartilage. This study investigated the early genetic responses of healthy *in situ* chondrocytes under IL-1β attack with a focus on cell cycle and calcium signaling pathways. RNA sequencing analysis identified 2,232 significantly changed genes by IL-1β, with 1,259 upregulated and 973 downregulated genes. Catabolic genes related to ECM degeneration were promoted by IL-1β, consistent with our observations of matrix protein loss and mechanical property decrease during 24-day *in vitro* culture of cartilage explants. IL-1β altered the cell cycle (108 genes) and Rho GTPases signaling (72 genes) in chondrocytes, while chondrocyte phenotypic shift was observed with histology, cell volume measurement, and MTT assay. IL-1β inhibited the spontaneous calcium signaling in chondrocytes, a fundamental signaling event in chondrocyte metabolic activities. The expression of 24 genes from 6 calcium-signaling related pathways were changed by IL-1β exposure. This study provided a comprehensive list of differentially expressed genes of healthy *in situ* chondrocytes in response to IL-1β attack, which represents a useful reference to verify and guide future cartilage studies related to the acute inflammation after joint trauma.

## Introduction

Interleukin 1β (IL-1β) is an essential mediator of acute joint inflammation after traumatic injuries, one of the potential causes of post-traumatic osteoarthritis (PTOA). Within 24 hours after trauma injuries, the concentration of IL-1β in synovial fluid can increase up to 70 times to 140 pg/mL in human^1,2^ and 7 times to 6 ng/mL in mice^3^. Overexpression of IL-1β protein is also observed in chondrocytes of early osteoarthritic cartilage^4,5^. High level of IL-1β aggravates the catabolic activities of synovial cells and chondrocytes^6,7^ and stimulates chondrocytes to enter an abnormal phenotypic shift, such as proliferation of pre-chondrocytes, swelling of mature chondrocytes, and hypertrophic differentiation of cells in deep zone^8,9^. Associated with these changes are the increased release of enzymes from chondrocytes, such as the MMP (matrix metalloproteinases) and ADAMTS (a disintegrin and metalloproteinase with thrombospondin motifs) families. Thus acute inflammatory attack often results in the degeneration of healthy cartilage^7,10^.

Due to its important role in OA pathology, IL-1β-treated articular cells or tissues have been widely adopted as *in vitro* models to study OA initiation or PTOA^6,11^. For both isolated and *in situ* chondrocytes, IL-1β has been observed to induce transient concentration changes of intracellular calcium ([Ca^2+^]_i_) and small GTPases. The coordination of Rho GTPases signaling and [Ca^2+^]_i_ signaling plays a fundamental role in cytoskeleton organization, regulating the chondrocyte phenotypic shift and cartilage ECM homeostasis^8,9^. Many of these studies focused on specific genes/pathways in chondrocytes. A systematic study to clarify the effects of IL-1β on the entire gene expression profile and signaling transduction coordination remains lacking. In this study, we put special focus on the healthy chondrocytes under intensive inflammatory cytokine attack, which is a common scenario during acute inflammation phase after traumatic injuries, such as ACL rupture or meniscus tear in the knee joint.

The objective of this study is to obtain a complete list of gene expression changes in the healthy chondrocytes that are subjected to acute inflammatory attack. To maintain the natural environment of chondrocytes and perform longitudinal evaluation of cells and ECM, fresh cartilage explants were cultured *in vitro* and treated with IL-1β. We performed RNA sequencing analysis on the treated chondrocytes, as well as the enrichment analysis in KEGG curated pathways. To verify and understand the changes in genetic profiles, we also tracked the loss and synthesis of ECM components longitudinally, the poroelastic properties of ECM, the proliferation of cells, the change of cell volume and calcium signaling of *in situ* chondrocytes.

## Results

### Cartilage Degradation Induced by IL-1β

**Figure 1:**
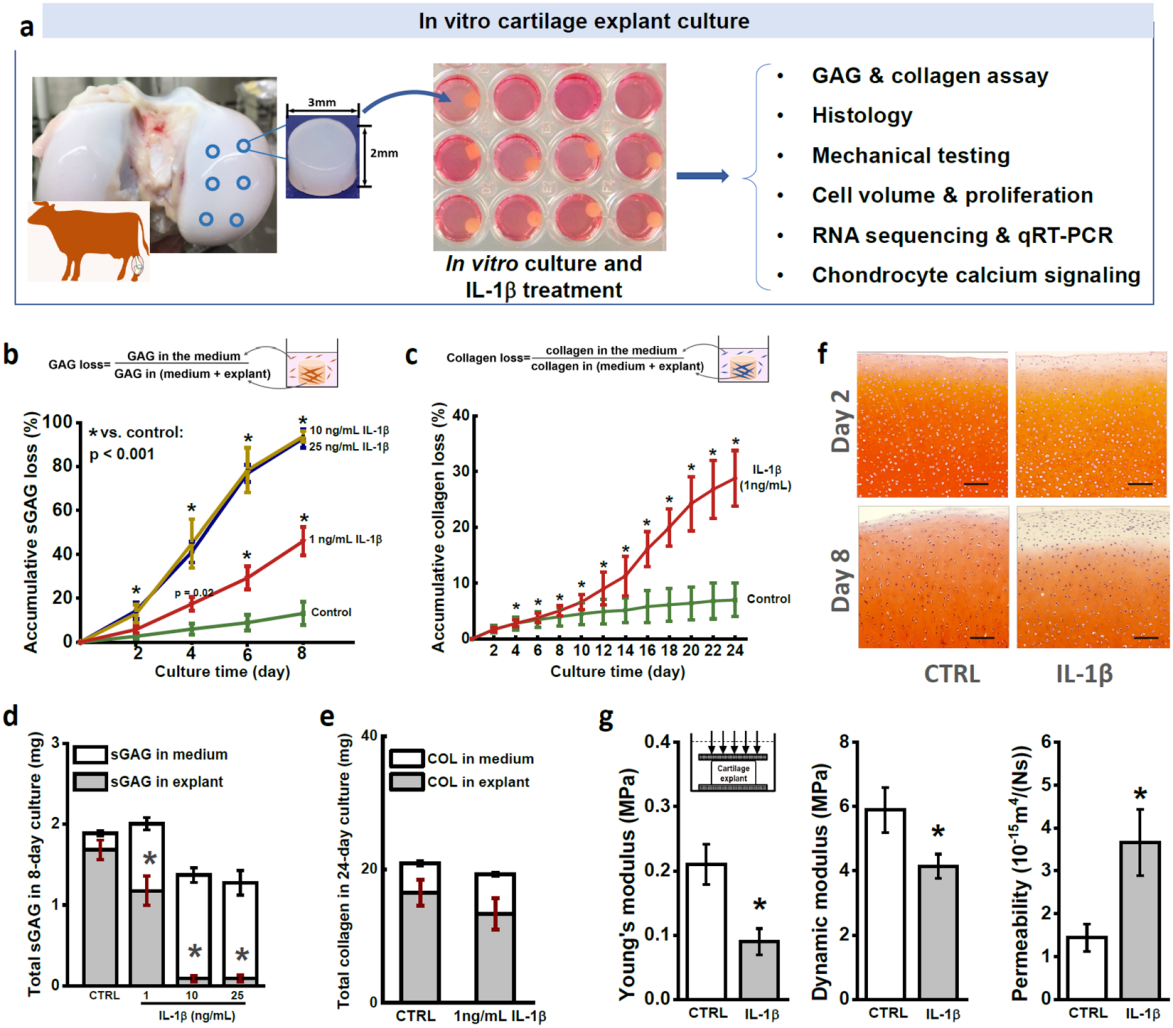
Effects of IL-1β during the long-term *in vitro* culture of cartilage explants. (**a**) Schematic diagram of experimental design. Cylindrical cartilage explants (diameter = 3 mm, thickness = 2 mm) were harvested from the central region of femoral condyle head of bovine knee joint using a biopsy punch. (**b**) Accumulative loss of sGAG content from cartilage explants during 8-day treatment of different doses of IL-1β. The accumulative sGAG loss was defined as the total sGAG released into the culture medium divided by total sGAG content in explant and culture medium. (**c**) Accumulative collagen loss from cartilage explant in 24-day treatment of 1 ng/mL IL-1β. (**d**) The total sGAG content of cartilage explants, which includes the sGAG released into medium (white bar) and sGAG left in explants (grey bar) at the end of 8-day culture. (**e**) The total collagen content and distribution of collagen in medium and explant after 24-day culture. (**f**) Safranin O staining of the superficial zone of cartilage explant after 2- and 8-day culture. Scale bar = 200 µm. (**g**) Mechanical properties of cartilage explants after 8-day IL-1β treatment, including equilibrium Young’s modulus, dynamic modulus, and permeability. All data were shown as mean ± 95% confidence intervals. * vs control, p < 0.001 if otherwise marked.

To assess the direct effects of IL-1β on cartilage structure integrity, we tracked the ECM contents loss using an *in vitro* culture cartilage explant model (Fig. 1a). Cylindrical cartilage explants were harvested from femoral condyle head of calf knee joints and cultured in chemically defined medium up to 24 days. IL-1β was supplemented into the culture medium, inducing the loss of sGAG content from cartilage explant in a dosage- and time-dependent manner (Fig. 1b). After 8-day treatment, IL-1β at concentration of 10 ng/mL and 25 ng/mL resulted in greater than 90% sGAG loss from the cartilage respectively (Fig. 1b). In contrast, 1 ng/mL IL-1β induced a stable, almost linear sGAG loss accumulating up to 50% on day 8 (46.0 ± 6.4% vs. 13 ± 1.2% in the control group) (Fig. 1b). According to these data, we adopted 1 ng/mL IL-1β treatment in the following tests. In mouse knee joint, the concentration of IL-1β can reach 6 ng/mL after traumatic damage^3^. IL-1β concentration of 1 ng/mL has been adopted widely in previous studies using animal cartilage samples^12,13^. Loss of collagen content induced by 1 ng/mL IL-1β remained at a low rate on the first 8 days and significantly accelerated afterwards (Fig. 1c). After 24 days, collagen loss accumulated to 32.7 ± 4.3% (control: 4.9 ± 0.5%). IL-1β at 1 ng/mL showed no significant effects on the sGAG or collagen synthesis rate of the *in situ* chondrocytes (Fig. 1d,e).

Safranin O staining revealed the spatial pattern of sGAG loss across the cartilage explant (Fig. 1f). After mere 2-day IL-1β treatment, sGAG loss can be noticed in the surrounding areas of the treated explant. The difference between the control and treated samples became even more evident on day 8. Loss of sGAG content showed a sharply increasing gradient along the center to edge direction. Such inhomogeneous sGAG loss pattern can significantly compromise the tissue stiffness at small strain, as the outer layer with low sGAG content has little resistance to compression and will be easily compressed under small loading. Mechanical properties of cartilage explants reflected the degradation and loss of ECM components. After 8-day IL-1β treatment, both Young’s modulus (treated vs. control: 0.09 ± 0.02 vs. 0.21 ± 0.03 MPa) and dynamic modulus (4.14 ± 0.38 vs. 5.89 ± 0.7 MPa) decreased significantly, while the hydraulic permeability of the tissue increased by over 150% (treated vs. control: 3.71 ± 0.81 × 10^−15^ vs. 1.43 ± 0.32 × 10^−15^ m^4^/N·s) (Fig. 1g). No significant differences in mechanical properties were detected after 2-day IL-1β treatment (Supplementary Fig. 1).

### Differentially Expressed Genes (DEGs)

**Figure 2:**
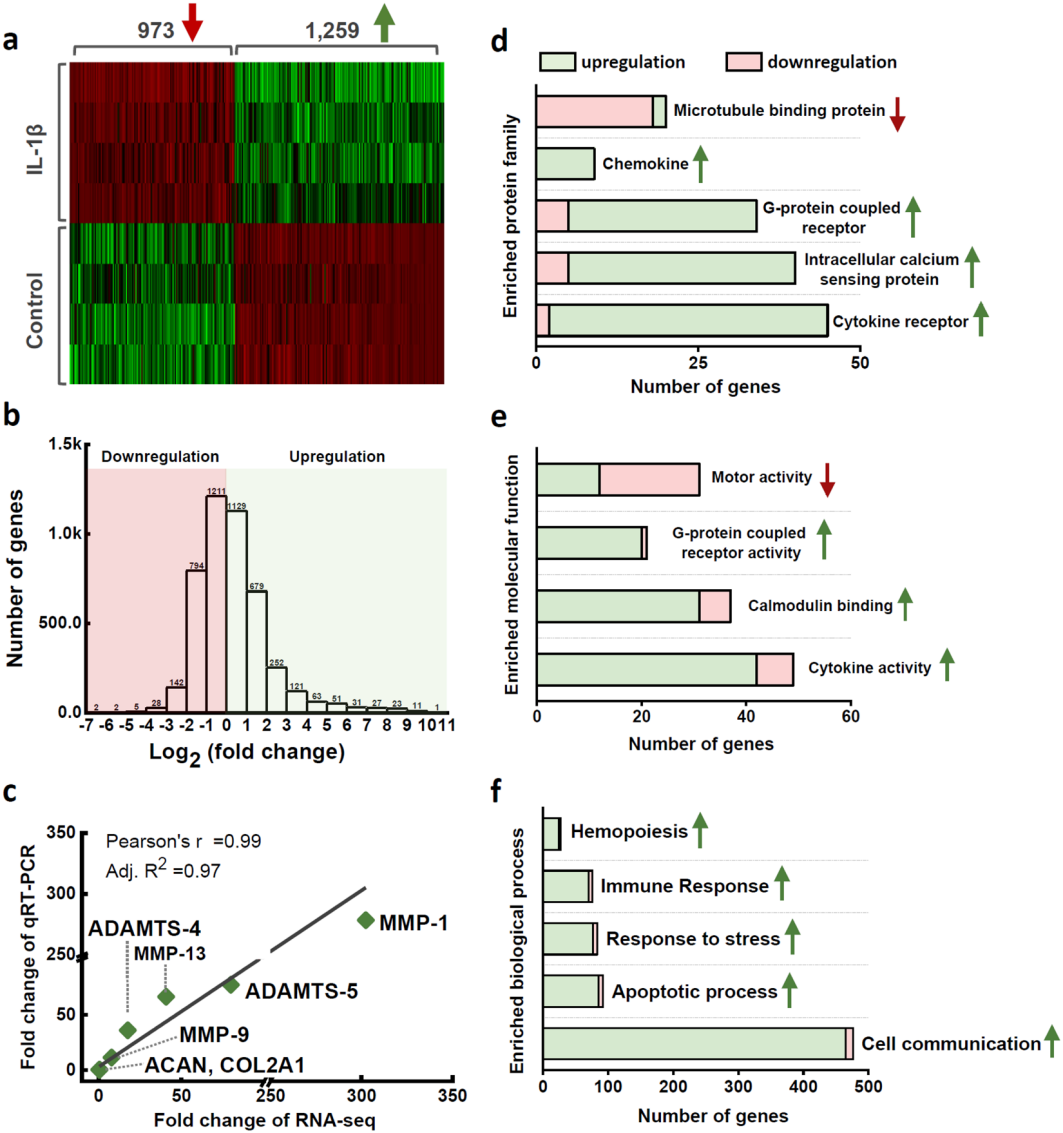
Summary statistics of RNA sequencing data. (**a**) Unsupervised hierarchical clustering was performed for differentially expressed genes (absolute value of fold change >2 and FDR < 0.05) in the IL-1β treated cartilage explants. (**b**) Distribution of gene expression fold changes according to log_2_ transformation. (**c**) Validation of RNAseq results with qRT-PCR. Seven selected genes were tested and correlated with the Pearson’s correlation analysis. (**d**) Enrichment analysis of the protein family classification and Gene Ontology annotations in terms of (**e**) molecular functions and (**f**) biological processes.

RNA sequencing was used to assess the early gene expression profile of *in situ* chondrocytes under the stimulation of IL-1β (a full gene list in Supplementary Data). Compared to the healthy control, IL-1β treatment induced 2,232 DEGs (absolute value of fold change > 2 and FDR < 0.05) in the chondrocytes, among which 1,259 genes were upregulated (fold change = $\frac{{\rm{expression}}\,{\rm{level}}\,{\rm{in}}\,{\rm{IL}}-1{\rm{\beta }}}{{\rm{expression}}\,{\rm{level}}\,{\rm{in}}\,{\rm{control}}}$) and 973 genes downregulated (fold change = $-\frac{{\rm{expression}}\,{\rm{level}}\,{\rm{in}}\,{\rm{control}}\,}{{\rm{expression}}\,{\rm{level}}\,{\rm{in}}\,{\rm{IL}}-1{\rm{\beta }}}$). The heat map of DEGs is composed of four sharply isolated blocks that indicated the prominent effects of IL-1β on chondrocyte transcriptional profiles (Fig. 2a). Fold changes of all the genes were analyzed in Fig. 2b. In total, 12,655 genes were mapped, with 17.6% DEGs. The fold changes of MMP-1, MMP-9, MMP-13, ADAMTS-4, ADAMTS-5, ACAN, and COL2A1, determined by the qRT-PCR and RNA sequencing were highly consistent, with a Pearson’s R value of 0.99 and an adjusted R^2^ of 0.97 (Fig. 2c). IL-1β-induced expression changes in catabolic genes (MMPs and ADAMs) were hundreds of folds and much higher than the changes (a few folds) of anabolic genes (COL2 and ACAN)^12,14^.

### Matrix Protein Related DEGs

According to previous studies, we summarized the cartilage-specific biological function of each gene from collagen, proteoglycan, MMP and ADAMTS family in Supplementary Table 1. The expression levels of collagen of type II (COL2A1), type IX (COL9A1, 2, 3), and type XI (COL11A1, 2) were significant in the healthy cells with RPKM over 10,000 (for reference, RPKM of actin: 1137.52). Expression changes of COL9 and COL11 were decreased by more than 2 folds by IL-1β, and COL2 was decreased by 1.82 folds (FDR < 0.05, RPKM of control: 224154.47). The pericellular matrix (PCM) genes, COL6A1 (3.89 folds) and COL6A2 (4.03 folds) were promoted; while the perlecan (HSPG2) and COL6A3 (the most abundant COL6 gene) were not significantly altered by IL-1β. Only three proteoglycan related genes were significantly changed: aggrecan (ACAN: −2.33 folds, RPKM of control: 55400.32), hyaluronan (HAS2: −2.49 folds, RPKM of control: 81.56) and decorin (DCN: 2.68 folds, RPKM of control: 336.41). No significant changes were detected in other proteoglycans, *e.g*., biglycan (BGN, RPKM: 6514.63), fibromodulin (FMOD, RPKM: 7487.12), lumican (LUM, RPKM: 254.16) or perlecan (HSPG2, RPKM: 2399.10). The expression changes of cartilage growth factors that regulate the chondrocyte anabolic activities were summarized in Supplementary Table 2.

Expression levels of MMPs could vary between species^15^. Our data suggested that IL-1β promoted the expression of MMP-3 (188.78 folds), MMP-9 (6.77 folds), and MMP-13 (41.48 folds) in bovine chondrocytes. MMP-1, which is essential for the pre-processing of pro-collagens, was also changed by 317.73 folds, but its RPKM value in the control was as low as 0.19. MMP-28, which maintains cell adhesion to ECM network, was downregulated by 2.5 folds. MMP-2, 14 and 16, which maintain the routine cartilage ECM remodeling, were highly expressed in bovine chondrocytes but not changed by IL-1β (absolute value of folds change < 2 or FDR > 0.05). The most upregulated ADAMTSs included ADAMTS-4 (16.37 folds), ADAMTS-5 (82.62 folds), and ADAMTS-7 (12.29 folds). IL-1β decreased the expression of ADATMS-3 and ADAMTS-14, which play essential roles in processing the precursor of type II collagen for fibril formation^16^. ADAMTSL-4, a barely studied gene in chondrocytes, was downregulated by 12.71 folds by IL-1β and showed the highest mRNA level (RPKM: 106.97) among the ADAMTS family.

### Enrichment Analysis of Gene Ontology and Pathway

To understand the functions and interconnections of the 2,232 DEGs, enrichment analysis in terms of protein family classification and Gene Ontology (GO) annotation was performed. Gene family definition and a gene’s function are described using GO annotation based on the experimental findings in literature. The enriched protein families included microtubule binding protein, chemokine, G-protein coupled receptor, intracellular calcium sensing protein, and cytokine receptor (Fig. 2d). The enriched molecular functions, including G-protein coupled receptor activity, calmodulin binding, and cytokine activity, were promoted; while the motor activity was mainly inhibited (Fig. 2e). In the enriched biological process families, hemopoiesis, immune response, stress response, apoptotic process, and cell communication were all significantly upregulated (Fig. 2f).

**Figure 3:**
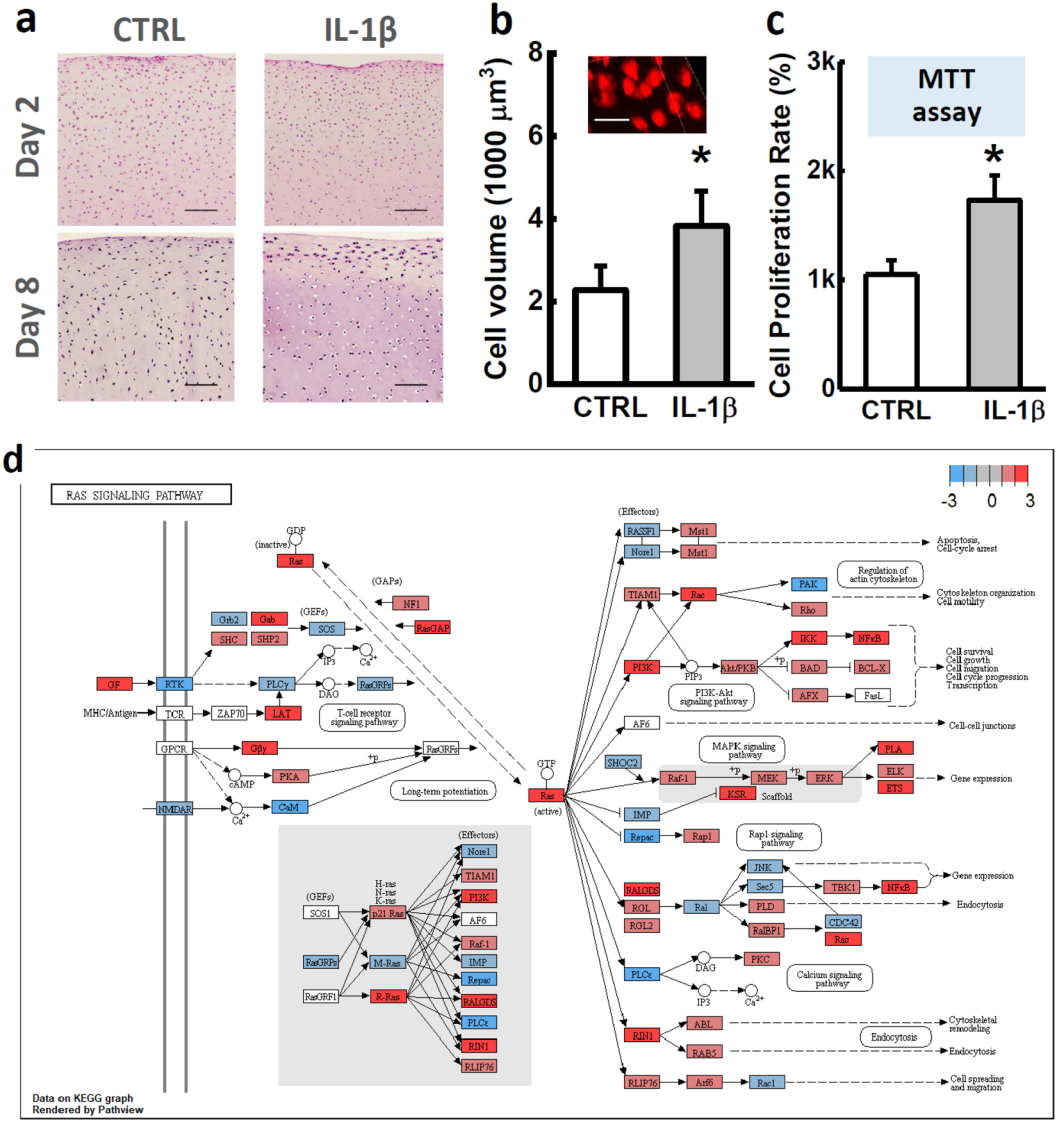
Chondrocyte swelling and proliferation. (**a**) H&E staining of cartilage explants after 2- and 8-day IL-1β treatment. Cell swelling, also termed as fattening, and proliferation can be observed after 8-day IL-1β culture. Scale bar = 200 µm. (**b**) Cell volume of chondrocytes in cartilage explants measured by 3D confocal imaging. Scale bar = 30 µm. (**c**) Cell proliferation rate determined by MTT assay. (**d**) Visualization of Ras superfamily signaling pathway regulated by IL-1β-treatment. Flowchart was plotted using RNAseq data in Pathview (version 3.6). Each node represented a gene, and the associated node color represented the expression changes in log2 ratios. For better readability, multiple genes with similar or redundant functional roles were pooled together as a single node. Absolute value of the maximum of expression changes of these genes was reflected by the node color. All data were shown as mean ± 95% confidence intervals. * vs control, p < 0.001 if otherwise marked.

The pathway enrichment analysis identified 20 IL-1β-changed pathways. For each pathway, the number of related DEGs and the associated P value were reported (Table 1). The cell cycle pathway was interrupted by IL-1β, which was confirmed by our experimental observations. In the H&E staining images, proliferation of pre-chondrocytes was obvious in the superficial zone, where many cells aggregated into cell clusters. In the center of explant, IL-1β induced a significant cell volume increase (Fig. 3a). Confocal imaging showed 68% cell volume increase in the IL-1β treated group (control vs. IL-1β, 2.27 ± 0.59 × 10^3^ vs. 3.83 ± 0.84 × 10^3^ µm^3^) (Fig. 3b). MTT assay showed that the proliferation rate of the primary chondrocytes doubled in the IL-1β-supplemented Medium (Fig. 3c). RNAseq revealed that IL-1β suppressed the negative regulators of chondrocyte hypertrophy, including insulin-like growth factor (IGF2: −3.36 folds), pappalysin (PAPPA: −2.49 folds; PAPPA2: −4.52 folds), and IGF-binding protein (IGFBP2: −5.41 folds). Plate derived growth factor (PDGF) pathway can promote the chondrocyte proliferation^17^, among which 55 related genes were significantly changed, such as PDGFC (16.76 folds) and PDGFD (3.67 folds) (Table 1).

**Table 1:** Pathway enrichment analysis identified 20 child pathways (sub level 2 to 3) that are significantly changed by IL-1β.

Group	Reactome pathway	Gene number	FDR
Cell cycle	Cell cycle, mitotic	108	0.00
	Chromosome maintenance	23	0.00
	G2/m checkpoints	21	0.02
Cell-cell communication	Cell-cell communication	26	0.06
Cellular responses to stress	Senescence-associated secretory phenotype	14	0.10
DNA replication	DNA replication	26	0.03
ECM organization	Degradation of the extracellular matrix	33	0.00
	Collagen formation	28	0.00
	Integrin cell surface interactions	19	0.02
	Elastic fiber formation	13	0.02
	ECM proteoglycans	15	0.03
	Laminin interactions	9	0.05
	Non-integrin membrane-ECM interactions	12	0.06
Immune system	Toll-like receptors cascades	37	0.00
	Cytokine signaling in immune system	95	0.00
Metabolism	Glycosaminoglycan metabolism	26	0.03
Metabolism of proteins	Regulation of IGF transport and uptake by IGF binding proteins	8	0.03
Programmed cell death	Caspase activation via extrinsic apoptotic signaling	10	0.03
Signal transduction	Rho GTPases signaling	72	0.00
	PDGF signaling	55	0.08

The child pathways of FDR < 0.1 were selected and grouped together if belonging to one parent (top-level, left column) pathway.

The course of cell cycle is highly correlated with cytoskeleton metabolism^18^. A summary of all cell cycle related DEGs was provided in Supplementary Fig. S2 (a). Rho/Ras GTPases are essential “molecular switches” for the cytoskeleton organization of chondrocytes^8,19^. Expression change of Rho/Ras signaling related genes was summarized in the flow chart of Fig. 3d, where each node represents a gene, and node color indicates the expression changes revealed by our RNAseq. If multiple genes with similar or redundant functional roles were pooled together as a single node, the node color was indicated by the absolute value of the maximum expression changes of these genes. In Rho/Ras signaling, 72 genes were changed significantly, such as Ras-related GTPase effector (RALGDS: 9.37 folds), Ras homolog family gene (RHOQ: 2.92 folds), Ras-Rab interactor (RIN1: 5.28 folds), R-Ras gene (RRAS: 2.31 folds), and Rho GTPase activating protein (ARHGAP10: 2.91 folds). These data revealed the important role of Rho/Ras GTPases in the mediation of the catabolic signaling pathways in chondrocytes.

IL-1β changed the expression of 95 (19.4%, 489 in total genes) genes in cytokine signaling pathway and 37 (28.0%, 132 in total) genes in toll-like receptors (TLRs) cascades pathway. The basal expression levels of cytokine genes in healthy chondrocytes were low (RPKM: 0.03–5.24), while IL-1β significantly increased the expressions of IL1B, IL6, IL34 by >30 folds. A central pro-inflammatory regulator, NF-κB, was significantly promoted (NFKB2: 7.11 folds; and NFKB1: 4.46 folds). TLR2, 3, 4, 6, 7, and 10 showed similar basal expression levels in healthy chondrocytes. TLR2 (160.66 folds, RPKM: 1.49) and TLR4 (6.14 folds, RPKM: 1.72) were upregulated by IL-1β, similarly as reported in literature^20,21^. In addition, eight essential pathway networks (*e.g*., cell cycle, autophagy, chemokine signaling, and JAK-STAT signaling) with gene expression changes were presented in Supplementary Fig. S2, as well as their specific roles in cartilage metabolism and OA pathology.

### Intracellular Calcium Signaling

**Figure 4:**
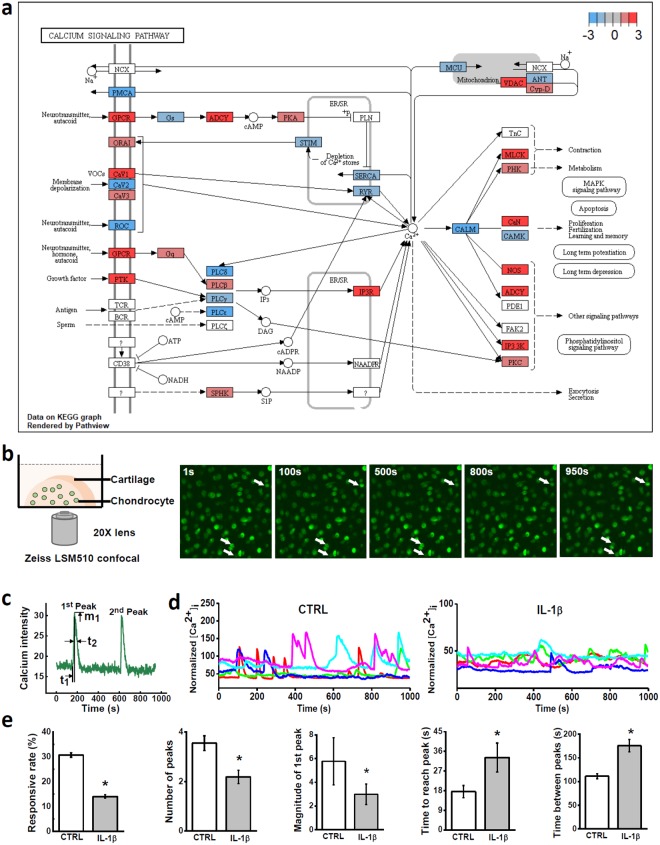
Calcium signaling of chondrocytes in cartilage explants. (**a**) Visualization of calcium signaling pathway regulated by IL-1β. (**b**) Illustration of calcium signaling. Half cartilage explant was dyed with fluorescent indicator and imaged on a confocal microscope for 15 minutes. Due to the fluctuation of [Ca^2+^]_i_ concentration, image intensity of chondrocyte oscillates in recorded video (see supplementary material for calcium signaling video). (**c**) Definition of spatiotemporal parameters of [Ca^2+^]_i_ peaks. (**d**) Representative [Ca^2+^]_i_ curves of *in situ* chondrocytes from the control group and IL-1β treated group. (**e**) Parameters of [Ca^2+^]_i_ peaks, including the average number of [Ca^2+^]_i_ peaks, magnitude of peaks, time to reach a peak, and time between neighboring peaks. All data were shown as mean ± 95% confidence intervals. * vs control, p < 0.05 if otherwise marked.

Using our RNAseq data, we specifically looked into a fundamental pathway, [Ca^2+^]_i_ signaling pathway, which regulates a wide range of biological processes, such as cell metabolism and morphology (Fig. 4a). The expression changes of 24 genes related to 6 calcium-signaling pathways were listed in Table 2. Transient receptor potential vanilloid 4 (TRPV4) was substantially expressed in healthy chondrocytes and promoted by 1.31 folds by IL-1β (RPKM: 159.77, FDR < 0.05), while TRPV1, 2, and 3 were barely expressed in bovine chondrocytes (RPKMs < 1). Piezo-type mechanosensitive ion channel, PIEZO1 and PIEZO2, had similar mRNA levels in chondrocytes (RPKM: 107.22 and 152.53). PIEZO1 was upregulated by 4.11 folds by IL-1β, with PIEZO2 unchanged (−1.13 folds). Voltage-Gated Calcium Channels (VGCCs) were significantly regulated by IL-1β, including N-type channel (CACNA1B: −15.34 folds, RPKM: 2.44), R-type channel (CACNA1E: −3.13 folds, RPKM: 11.72), and L-type channel (CACNA1C: 5.2 folds, RPKM: 4.13). T-type VGCC (CACNA1H) had no significant change despite its abundant mRNA level in chondrocytes (1.04 folds, 121.86 RPKM). A VGCC auxiliary subunit, CACNA2D1, was the most expressed VGCC gene in chondrocytes and suppressed by IL-1β (−1.72 folds, RPKM: 232.39).

**Table 2:** Summary of key genes involved in six calcium-related pathways.

Calcium-related pathway	Gene symbol	RNAseq gene expression				
		Fold change (IL-1β/CTRL)		RPKM (CTRL)	RPKM (IL-1β)	FDR
Mechano- sensitive channel	TRPV4	+	1.31	159.77	209.80	0.09
	PIEZO1	+	4.11	107.22	440.92	0.00
Ligand-gated ion channel	GRIN2C	+	2.19	0.67	1.47	0.06
	GRIN2D	+	2.29	10.32	23.61	0.00
	GRINA	+	2.59	21.95	56.82	0.00
Voltage-sensitive calcium channels	CACNA1B	—	15.34	2.44	0.16	0.00
	CACNA1E	—	3.13	11.72	3.75	0.03
	CACNA2D1	—	1.72	232.39	135.42	0.00
	CACNA1C	+	5.20	4.13	21.44	0.00
Purinergic receptor	P2RX6	—	3.69	1.88	0.51	0.00
	P2RX4	—	1.43	20.30	14.21	0.03
	P2RY2	—	1.36	12.66	9.28	0.06
	P2RX5	+	1.97	4.95	9.73	0.00
PLC-IP_3_	PLCH1	—	14.64	3.08	0.21	0.00
	PLCD1	—	2.29	297.00	129.71	0.00
	PLCE1	—	2.26	179.13	79.20	0.00
	PLCD3	+	2.27	4.75	10.76	0.00
	PLCH2	+	2.29	1.08	2.48	0.02
	PLCH2	+	2.29	1.08	2.48	0.02
	ITPRIP	+	3.37	8.19	27.62	0.00
	ITPR3	+	6.35	0.79	4.99	0.00
ER store	STIM2	—	1.64	41.68	25.43	0.00
	STIM1	+	1.31	14.45	18.89	0.10
	ORAI2	+	1.95	2.96	5.76	0.01

The “−” represents downregulation, and “+” represents upregulation.

To evaluate the ultimate effects of IL-1β on chondrocyte [Ca^2+^]_i_ signaling, we recorded the spontaneous [Ca^2+^]_i_ signaling of *in situ* chondrocytes after 2-day IL-1β treatment (Fig. 4b). Typical [Ca^2+^]_i_ oscillations of *in situ* chondrocytes were shown in Fig. 4c,d and and videos in Supplemental Movie. 203 out of 661 (30.7 ± 0.14%) cells showed spontaneous [Ca^2+^]_i_ oscillation in the control explants, a significantly higher responsive rate than that in the IL-1β treated samples (87 out of 621 cells, 14.0 ± 0.11%) (Fig. 4e). For each responsive cell, the spatiotemporal parameters of [Ca^2+^]_i_ peaks were also significantly altered by IL-1β. The magnitude of [Ca^2+^]_i_ peaks was reduced (control vs. IL-1β: 5.78 ± 1.01 vs. 2.98 ± 0.87), and the number of multiple peaks was decreased (control vs. IL-1β: 3.55 ± 0.3 vs. 2.19 ± 0.27). The time between two neighboring peaks (control vs. IL-1β: 111.26 ± 5.12 vs. 175.70 ± 13.24) and the time to reach a peak from baseline (control vs. IL-1β: 17.58 ± 2.78 vs. 33.2 ± 6.62) were both significantly prolonged by IL-1β, indicating slow Ca^2+^ transport (Fig. 4e).

## Discussion

Previous studies have attempted to evaluate the large-scale gene expression profile of chondrocytes using various OA models, including monolayer chondrocytes^13,22^, cartilage of human knee joints^23,24^, and cartilage from OA animal models^14,25^. An important finding of these studies is that chondrocytes present declined transcriptomic changes along with OA progression. Chondrocytes demonstrate drastic genetic changes before cartilage shows visible degradation, whilst they adapt to the degenerated extracellular environment with little chondrogenic features at the late stage of OA^5,24^. For instance, cartilage cells at late OA stage barely express catabolic genes, such as MMPs and ADAMTSs^24^. A detailed comparison between our RNAseq data and that of late-stage OA cartilage found only 214 (9.26%) genes are sharing the same trend (upregulation/downregulation)^24^. In contrast, high consistency was observed between our RNAseq data and those from chondrocytes at early-stage OA. Our study has 74% consistency with a microarray study of healthy human chondrocytes subjected to 96-hour IL-1β treatment^13^ and 67% consistency with a study of early degenerative human cartilage^26^. A full comparison lists between our RNAseq data and these two studies were provided in Supplementary Tables 3,4. There was 48% consistency with another gene expression profile of a middle-stage OA model (rats at 4 weeks post ACL rupture, 722 DEGs)^25^. To the best our knowledge, our RNAseq outcome is also highly consistent with a number of specific chondrocyte pathway studies, *e.g*., NFAT^27^, EGFR^28^, IGF^12,19,29^, and TRPV^30^ pathways. Therefore, a major contribution of our RNAseq study is to generate a complete list of genes of chondrocyte in response to acute joint inflammation. This study identified 2,232 DEGs and 10,423 non-DEGs, with information about each gene’s basal expression level at healthy status and the relative expression fold change in response to the IL-1β stimulation. The expression changes identified by the RNAseq are highly consistent with those reported in specific pathway or cartilage inflammation mechanism studies. Thus the present RNAseq data set can serve as a unique source to verify individual gene or pathway studies and provide guidance for future PTOA research.

In cartilage ECM, aggrecan, the major proteoglycan, forms supramolecular aggregates with hyaluronan and is entrapped in the collagen II/IX/XI fibrillar network^31^. The nonaggregating proteoglycans, *i.e*., fibromodulin, biglycan, decorin and lumican, can bind with various types of collagens and regulate the formation of fibril networks^32^. This specialized structure endows cartilage with its biomechanical properties for joint loading. In our RNAseq data, IL-1β significantly reduced the gene expression of aggrecan (ACAN) and hyaluronan (HAS2); however, the expression of type II collagen (COL2A1), fibromodulin (FMOD), biglycan (BGN), and lumican (LUM) showed no significant changes. These results corroborate previous observation that the degradation of proteoglycan aggregates is prior to that of collagen network at the early stage of OA initiation^12,32^. Decorin, another important nonaggregating proteoglycan, is known as an important regulator of collagen fibril and proteoglycan assembly^32^. Upregulation of decorin was observed in our RNAseq data as well as previous early-OA studies^33^. This response has been regarded as an attempt by chondrocytes to increase the adhesion between fragmented aggrecans, thereby delaying its loss from cartilage. During OA progression, the PCM of chondrocytes, which is mainly composed of type VI collagen and perlecan, also presents aberrant remodeling process^34^. The drastic PCM degradation is associated with and may be responsible for the phenotypic shift of chondrocytes. In the early OA stage, type VI collagen is increasingly expressed although its fibrils is disorganized with compromised density^35^. Our RNAseq data also showed the upregulation of all three collagen VI isoforms (COL6A1, 2, and 3) in the IL-1β-treated cartilage samples. Perlecan, co-localized with type VI collagen in the PCM, plays an important role in regulating chondrocyte anabolic activities. The retention of growth factors by perlecan, such as fibroblast growth factor (FGF2) and bone morphogenetic proteins (BMP2/7), has been shown to promote chondrogenic differentiation and matrix production^35^. WARP (von Willebrand factor A domain-related protein), a newly identified component of chondrocyte PCM, can also interact with perlecan to contribute to the assembly and maintenance of cartilage structures during cartilage development^36^. In our RNAseq result, neither perlecan (HSPG2) or WARP (VWA1) genes was significantly changed by the IL-1β treatment, indicating no drastic changes of their synthesis at the acute inflammation stage. Taken together, these ECM and PCM proteins may play synergetic but unique roles in regulating cartilage homeostasis, thus presenting distinct responses to acute inflammation stimulation.

An important finding from the present study was that IL-1β can induce significant changes in the [Ca^2+^]_i_ signaling of chondrocytes. As illustrated in Fig. 4a, [Ca^2+^]_i_ signaling can be activated partially through G-protein coupled receptors (GPCRs), an important mediator regulating chondrocyte morphology. In both proliferating and hypertrophic chondrocytes, the expression of GPCRs and regulators of G-protein signaling (RGS) increases markedly^22,37^. According to RNAseq data (Fig. 4a), the GPCR- and RGS-family genes were differentially regulated by IL-1β, such as GPR84 (133.07 folds), GPR68 (−4.81 folds), RGS8 (80.49 folds), and RGS22 (−5.31 folds), with parallel changes of downstream calcium-related genes including PLC- and adenylyl cyclase-(ADCY) family genes. Another initiation mechanism of [Ca^2+^]_i_ signaling is through the ion channels on plasma membrane. In chondrocytes, TRPV4 and PIEZO1 were recently identified as two key mechanosensitive ion channels. TRPV4 and PIEZO1 can be disturbed by inflammatory mediators, further inducing ECM degeneration^30,38,39^. According to our RNAseq data, TRPV4 and PIEZO1 were two highly expressed genes in healthy bovine chondrocytes, and both were changed by IL-1β with consistent trends as reported previously^30,39^. T-type VGCC, which exists mainly in excitable cells (*e.g*., neurons and muscle cells) to facilitate environmental calcium influx, plays an important role in regulating [Ca^2+^]_i_ signaling of chondrocytes under mechanical stimulation^40^. Our previous study showed that inhibition of T-type VGCCs can attenuate the OA-like phenotypes of chondrocytes by reducing the expression of mechanical-stress responsive genes Prostaglandin G/H synthase 2 (PTGS2) and osteopontin (SPP1)^41^. In this study, RNAseq detected minor expression change in T-type VGCC gene (CACNA1H), while both PTGS2 and SPP1 were promoted by 13.34 folds and 21.71 folds in the IL-1β-treated chondrocytes, respectively.

High level of IL-1β can shift chondrocytes towards an aberrant phenotype, including cell swelling, proliferation or hypertrophy. Rho GTPases, such as RhoA, Rac1, and Cdc42, are well recognized as crucial regulators of chondrocyte cytoskeleton and cell cycle^42^. IL-1β can affect the Rho family via [Ca^2+^]_i_ signaling, leading to the rearrangement of F-actin networks in chondrocytes^8,9^. Previous studies proved that overexpression of RhoA can suppress the ECM synthesis and chondrogenic differentiation of the chondrogenic cell line ATDC5^42,43^. Insulin-like growth factors (IGFs), an important anabolic factor in chondrocytes, can inhibit the abnormal Rho GTPases activities and therefore protect the cytoskeleton structure^12,19,29^. In our RNAseq data, IL-1β inhibited the IGF-modulated signaling pathway via suppressing IGF2 and IGF2R, with parallel decreases of PLCs genes (PLCH1, PLCD1, and PLCE1). Another growth factor that can activate Rho signaling is called platelet-derived growth factors (PDGFs), which is also involved in the chondrocytes proliferative process^17,44^. IL-1β promoted the PDGFC by 17 folds and PDGFD by 3.67 folds. Taken together, IL-1β-induced chondrocyte phenotypic shift is related to the Rho GTPases signaling, whose regulatory effects are related to IGF and PDGF.

A few limitations of this study should be noted. First, due to the limited access to healthy young human cartilage, bovine cartilage samples were used in this study. Nevertheless, this substitution can at least be partially justified by the greatest evolutionary conservation present between human and cattle^45^. Second, to avoid synergistic and interactive effects between cytokines, IL-1β was employed as the cytokine to simulate the inflammatory attack on cartilage during acute joint inflammation. Despite playing a central role in cartilage degeneration, IL-1β alone cannot recapitulate the complexity of multiple pro-inflammatory cytokines. A large number of cytokines are active in the joints, such as TNF-α, IL-1 and IL-6 families^6,7^. The genetic responses of chondrocytes revealed in the present study could vary from the actual situations in inflammatory joints. Third, chondrocytes’ reaction to cytokines is a highly time dependent behavior. This study focused on the effects of IL-1β on cartilage gene expression after 2-day treatment, whereas acute joint inflammation could last 2–4 weeks after traumatic injuries (*e.g*., meniscus tear). Fourth, investigation of either cell cycle or calcium signaling of chondrocytes could be complicated and have attracted tremendous efforts. Experiments in this study were designed as a verification of the ultimate effects of transcriptional changes induced by IL-1β. The experiments also functioned as case studies to illustrate the potential application of the RNAseq data in the verification and guidance of future OA studies related to the acute inflammation after joint trauma. For example, RNAseq data revealed that ACTB and GAPDH are proper reference genes for the genetic analysis of bovine chondrocytes (see Supplementary Table 5).

This study performed a detailed transcriptional analysis of IL-1β-treated healthy cartilage, which provides genetic evidence for the (1) short-term transcriptional responses and signaling transductions in chondrocytes during acute inflammation; and (2) the dysregulation of cell cycle and [Ca^2+^]_i_ signaling transductions of chondrocytes, both of which may play critical roles in modulating cell phenotypic shift under inflammatory attack. The comprehensive transcriptional profile identified here is highly consistent with other high-throughput studies and specific pathway studies in literature and thus may serve as useful guidance and verification for future chondrocyte research.

## Methods

### Effects of IL-1β on Cartilage Matrix and Chondrocytes

#### Cartilage explant harvest

Overall experimental design was outlined in Fig. 1a. Fresh young bovine knee joints (3–6 months old) were obtained from a local slaughter house (Green Village, NJ). Cylindrical cartilage explants (diameter = 3 mm, thickness = 2 mm) were harvested from the central, load-bearing region of femoral condyle head using a biopsy punch (Fig. 1a). After harvesting, samples were cultured in the chondrogenic medium (DMEM, 1% ITS + Premix, 50 μg/mL L-proline, 0.9 mM sodium pyruvate, 50 μg/mL ascorbate 2-phosphate) at 37 °C for 72 hours before further experiments^46,47^. After the balance to *in vitro* environment, cartilage explants were cultured in the medium supplemented with 1, 10, or 25 ng/mL bovine IL-1β recombinant protein (RBOIL1BI, Thermo Fisher) for 8 days. Matching samples from the same region on the condyle head were cultured in regular medium and served as the non-IL-1β treated control.

#### Loss of sGAG and collagen contents over time

During the *in vitro* culture of cartilage explants (n = 10 explants from 5 animals per group), the culture medium (500 µL/sample) was changed and collected every other day. The sGAG and collagen assay were performed as described previously^12,48^. The accumulative sGAG or collagen loss was calculated as the sGAG or collagen released into the medium divided by the sum of sGAG or collagen contents in both explant and culture medium^12^. According to the temporal features of IL-1β-induced ECM degradation from cartilage explant^12^, the sGAG loss was measured during the first 8-day culture, while the collagen loss was tracked for 24 days. Different cartilage explants were used to track the losses of sGAG and collagen, respectively.

#### Histology

After 2- and 8-day culture, histological analysis was performed on cartilage explants from the IL-1β treated and control groups (n = 2 explants from 2 animals per group). Explants were cut into 5-µm thick sections along depth direction and stained with Safranin O (Sigma) and Hematoxylin and Eosin Y (H&E, Sigma).

#### Cell swelling and proliferation

To evaluate the effect of IL-1β on chondrocyte volume, cartilage explants cultured in the regular medium and IL-1β-Supplemented Medium (n = 4 explants from 2 animals per group) were dyed with red fluorescent cell tracker (Red CMTPX Dye, Thermo Fisher) and imaged on a confocal microscope (Zeiss 510) after 4-day culture. The fluorescent image stacks were reconstructed into a 3D image in Image J^49^. The volume of *in situ* chondrocytes was registered and quantified (n ≈ 30 cells from each explant). To estimate the cell proliferation rate, primary chondrocytes were extracted from cartilage explants (n = 4 explants from 2 animals per group). MTT assay was then performed following the previous instructions^50^.

#### Mechanical properties

Unconfined compression test was used to longitudinally measure the mechanical properties of the cartilage explants at days 2 and 8 (n = 10 explants from 5 animals per group; samples cultured for mechanical testing only)^51^. During the test, a 10% strain was applied on the cartilage sample at a constant speed followed by a 20-min relaxation period. After the reaction force reached an equilibrium state, sinusoidal dynamic loading was applied on the sample for 15 minutes at 0.5 Hz with a magnitude of ±1%^52^. Equilibrium Young’s modulus and dynamic modulus of the samples were determined using the recorded force. Hydraulic permeability of the tissue was obtained by curve-fitting the stress relaxation curve using a nonlinear poroelastic model for cartilage^53,54^.

### RNA Sequencing Analysis and qRT-PCR of IL-1β Treated Cartilage

Cartilage explants were assigned into two groups and cultured in: 1) regular medium, and 2) 1 ng/mL IL-1β supplemented medium for 48 hours (n = 4 explants from 4 animals per group). The cellular RNA was extracted^55^. RNA samples with mass >2 μg and RIN score >6.5 were qualified for the following RNA sequencing and qRT-PCR tests. The sample size and quality test threshold were determined according to literature guidelines^56,57^.

RNAseq library of each sample was constructed from 1 μg of RNA using the TruSeq® Stranded mRNA Sample Preparation Kit (Illumina). Samples were pooled and sequenced on the Illumina HiSeq. 2500, and at least 13 million reads (51 bp single-end read) were generated for each sample. Data processing was performed using CLC Genomics Workbench v7.5 (QIAGEN). The low-quality sequence ends (>Q15), sequencing adapters, read with ambiguous nucleotides (>1 nucleotide), and short sequences (<40 bp after trimming) were removed before mapping to the bovine genome (version 4.6.1). Gene expression value was calculated as the total number of unique reads mapped to the exon sequence and normalized to reads per kilo base of transcript per million mapped reads (RPKM), and RPKM should correlate positively to the expression level of each gene. The differential expression of each gene was determined by the generalized linear model with animal as a random variable^58^. A stringent cutoff of False Discovery Rate (FDR)<0.05 and absolute fold change >2 was adopted to identify the differentially expressed genes (DEGs) between the IL-1β treated and the control samples.

Enrichment analysis of protein families and Gene Ontology annotations on DEGs data set was performed by Panther Classification System^59^. Enrichment analysis of pathways was performed using Reactome software^60^. To remove redundancy, only the enriched pathways from levels two to three defined by Reactome were reported and grouped by the level one parent pathway. The KEGG pathways generated by Pathview (version 3.6) were used to visualize the changes in a specific signaling pathway^61,62^.

To verify the RNA sequencing data, qRT-PCR was performed on the same RNA used for sequencing. Expression levels of seven major metabolic genes in chondrocytes were quantified, including aggrecan (ACAN), type II collagen (COL2A1), MMP-1, −9, −13 and ADAMTS-4, −5. Gene expression fold change was calculated using the 2(-Delta Delta C(T)) method after data normalized to the average of reference gene (β-actin, ACTB).

### Calcium Signaling of Chondrocytes

Spontaneous [Ca^2+^]_i_ signaling of *in situ* chondrocytes in cartilage explant was analyzed after 48-hour IL-1β treatment and compared to that of control. After the IL-1β treatment, cartilage explants (n = 5) were halved axially and dyed with 5 μM Fluo-8AM for 40 minutes^47,52^. Dyed sample was placed in an imaging chamber and mounted on a confocal microscope (Zeiss LSM510) (Fig. 4b). Calcium images of the *in situ* chondrocytes located in the center of cross section area were recorded every 1.5 seconds for 15 minutes. [Ca^2+^]_i_ signaling of chondrocytes was analyzed as we described previously^40,52^. The responsive percentage of cells was calculated as the fraction of cells with one or more [Ca^2+^]_i_ peaks over the total number of cells. The spatiotemporal parameters of the [Ca^2+^]_i_ peaks, including the number of multiple peaks, the magnitude of peaks, the time to reach a peak and the time interval between neighboring peaks, were also measured and compared.

### Statistical Analysis

Tukey’s Honestly Significant Difference test was performed following the one-way ANOVA to compare the sGAG loss induced by multiple IL-1β dosage. The Chi-square test was utilized to compare the responsive rate of [Ca^2+^]_i_ signaling. Student’s *t*-test was performed to compare all the other data between the IL-1β (1 ng/mL) and control groups. All data were shown as mean ± 95% confidence intervals. Statistical significance was indicated when P value < 0.05.

## Electronic supplementary material

Supplementary Information

Dataset 1

A video of typical calcium signaling of healthy cartilage

A video of typical calcium signaling of IL-1β-treated cartilage

## Data Availability

The datasets generated during and/or analyzed during the current study are available from the corresponding author on reasonable request. RNA sequencing data are available in Supplementary Files, and will be uploaded to NCBI together with the raw data after this work is accepted for publication.
