# Supplementary material for: Identification of Chondrocyte Genes and Signaling Pathways in Response to Acute Joint Inflammation
Source: Sci Rep. 2019 Jan 14;9:93. doi: 10.1038/s41598-018-36500-2 (PMC6331554; doi:10.1038/s41598-018-36500-2)
Supplement: Supplementary file 1 — Supplementary Information [file 41598_2018_36500_MOESM1_ESM.pdf]

Supplementary Information

Identification of Chondrocyte Genes and Signaling Pathways in Response to Acute Joint Inflammation

*Mengxi Lv[1,2], Yilu Zhou[2], Shawn W. Polson[1], Leo Q. Wan[3], Meiqing Wang[4], Lin Han[5], Liyun Wang[2], X. Lucas Lu[1,2, *]*

[1]Center for Bioinformatics and Computational Biology, University of Delaware, Newark, DE, United States

[2]Department of Mechanical Engineering, University of Delaware, Newark, DE, United States

[3]Department of Biomedical Engineering, Rensselaer Polytechnic Institute, Troy, NY, United States

[4]Department of Oral Anatomy and Physiology and TMD, the Fourth Military Medical University, Xi'an, Shanxi, China

[5]School of Biomedical Engineering, Science, and Health Systems, Drexel University, Philadelphia, PA, United States

For this manuscript, the Supplementary Information included:

- **4 Supplementary Table 1 – 4 (this file)**
- **2 Supplementary Figure S1 - S2 (this file)**
- **2 Supplementary Videos (2 separate wmv files)**
- **1 Supplementary Data (1 separate excel file)**

Supplementary Table 1. Major catabolic and anabolic genes in DEGs (absolute value of fold change > 2 and FDR < 0.05), including MMP- and ADAMTS-, collagen-, proteoglycan-family genes. The "-" in front of the fold change value represents downregulation of expression, and "+" represents upregulation of expression after IL-1β treatment. The RPKM was calculated to assess the abundance of transcript of each gene, which is correlated positively to the gene expression level.

Family	Gene symbol	RNAseq Gene Expression					Identified Biological functions
		Fold change		RPKM (CTRL)	RPKM (IL-1β)	FDR	
MMP	MMP28	-	2.50	21.81	8.71	0.00	regulate cell morphology [1]
	MMP23B	-	2.31	28.43	12.31	0.00	unknown
	MMP25	+	4.46	0.25	1.11	0.00	unknown
	MMP9	+	6.77	10.16	68.78	0.00	induce apoptosis of hypertrophic chondrocytes [2]
	MMP12	+	9.15	2.50	22.92	0.00	fetal development and malignant transformation [3]
	MMP13	+	41.48	4.80	199.14	0.00	degrade collagen [2]
	MMP3	+	188.78	15.32	2892.29	0.00	degrade a wide array of extracellular molecules [2]
	MMP1	+	317.73	0.19	60.54	0.00	degrade collagen types I, II, III [2]
ADAMTS	ADAMTS14	-	35.12	13.14	0.37	0.00	process procollagen for collagen fibril formation [4]
	ADAMTS6	-	6.59	88.10	13.37	0.00	unknown
	ADAMTS3	-	3.15	99.03	31.43	0.00	process procollagen for collagen fibril formation [4]
	ADAMTS20	-	2.80	2.56	0.92	0.01	unknown
	ADAMTS12	-	2.38	15.35	6.46	0.00	degrade cartilage oligomeric protein (COMP) [4]
	ADAMTS7	+	12.29	2.75	33.77	0.00	degrade cartilage oligomeric protein (COMP) [4]
	ADAMTSL4	+	12.71	106.97	1359.54	0.00	bind to fibrillin for microfibril formation [5]
	ADAMTS4	+	16.37	5.86	95.94	0.00	degrade major cartilage proteoglycan [4]
	ADAMTS5	+	82.62	0.20	16.40	0.00	degrade major cartilage proteoglycan [4]
Collagen	COL8A1	-	8.04	300.94	37.44	0.00	Unknown
	COL6A5	-	3.54	2.15	0.61	0.01	form Pericellular Matrix [6]
	COL9A1	-	3.47	14440.75	4164.15	0.00	decorate fibril surfaces of PCM [6]
	COL9A3	-	3.28	12160.36	3704.14	0.00	decorate fibril surfaces of PCM [6]
	COL11A1	-	3.16	31786.93	10070.64	0.00	synthesize as a heterotrimeric molecule in developing cartilage [6]
	COL9A2	-	2.94	10553.26	3587.21	0.00	decorate fibril surfaces of PCM [6]
	COL12A1	-	2.60	2015.97	775.16	0.00	bind to fibril surfaces but not covalently attached (FACIT collagen subfamily) [6]
	COL8A2	-	2.27	87.51	38.57	0.00	Unknown
	COL11A2	-	2.41	16250.45	6732.92	0.00	synthesize as a heterotrimeric molecule in developing cartilage [6]
	COL13A1	+	3.77	0.28	1.04	0.00	Unknown
	COL4A5	+	4.01	1.44	5.75	0.01	form Pericellular Matrix [7]
	COL6A1	+	3.89	2110.85	8217.24	0.00	form Pericellular Matrix [6]
	COL6A2	+	4.03	669.30	2696.74	0.00	form Pericellular Matrix [6]
	COL25A1	+	4.62	25.29	116.93	0.00	unknown
Proteo-glycan	ACAN	-	2.33	55400.32	23774.02	0.00	form the cartilage-specific proteoglycan core protein [8]
	DCN	+	2.68	336.41	900.56	0.00	control collagen fibrillogenesis [8]

1. Rodgers, U. R. *et al.* Expression and function of matrix metalloproteinase (MMP)-28. *Matrix Biol.* **28,** 263–272 (2009).
2. Rose, B. J. & Kooyman, D. L. A Tale of Two Joints: The Role of Matrix Metalloproteases in Cartilage Biology. *Disease Markers* (2016). doi:10.1155/2016/4895050
3. Kaspiris, A. *et al.* Macrophage-specific metalloelastase (MMP-12) immunoexpression in the osteochondral unit in osteoarthritis correlates with BMI and disease severity. *Pathophysiology* **22,** 143–151 (2015).
4. Yang, C. Y., Chanalaris, A. & Troeberg, L. ADAMTS and ADAM metalloproteinases in osteoarthritis – looking beyond the 'usual suspects'. *Osteoarthritis and Cartilage* (2017). doi:10.1016/j.joca.2017.02.791
5. Hubmacher, D. & Apte, S. S. ADAMTS proteins as modulators of microfibril formation and function. *Matrix Biology* (2015). doi:10.1016/j.matbio.2015.05.004
6. Eyre, D. Collagen of articular cartilage. *Arthritis Res.* **4,** 30–5 (2002).
7. Foldager, C. B. *et al.* Collagen Type IV and Laminin Expressions during Cartilage Repair and in Late Clinically Failed Repair Tissues from Human Subjects. *Cartilage* (2016). doi:10.1177/1947603515604022
8. Gallagher, J. T. The extended family of proteoglycans: social residents of the pericellular zone. *Curr. Opin. Cell Biol.* (1989). doi:10.1016/S0955-0674(89)80072-9

Supplementary Table 2. Summary of 21 important growth factors of cartilage [9]. The "-" in front of the fold change value represents downregulation of expression, and "+" represents upregulation of expression after IL-1β treatment.

Gene name	Gene name	Fold change	RPKM (CTRL)	RPKM (IL-1b)	FDR
**CTGF**	connective tissue growth factor	1.54	339.45	524.00	0.12
**BMP1**	bone morphogenetic protein 1	9.78	85.21	833.06	0.00
**BMP2**	bone morphogenetic protein 2	5.36	4.24	22.69	0.00
**BMP3**	bone morphogenetic protein 3	2.61	1.00	2.60	0.02
**BMP4**	bone morphogenetic protein 4	-1.10	1.50	1.36	0.88
**BMP5**	bone morphogenetic protein 5	-4.38	29.96	6.83	0.00
**BMP6**	bone morphogenetic protein 6	-1.05	33.04	31.49	0.98
**BMP7**	bone morphogenetic protein 7	120.56	0.17	20.00	0.00
**FGF11**	fibroblast growth factor 11	-1.12	16.48	14.77	0.93
**FGF14**	fibroblast growth factor 14	-1.15	3.34	2.90	0.63
**FGF18**	fibroblast growth factor 18	8.48	0.45	3.83	0.00
**FGF2**	fibroblast growth factor 2	3.30	4.92	16.25	0.00
**HDGF**	hepatoma-derived growth factor	-1.14	136.42	119.68	0.46
**IGF2**	insulin like growth factor 2	-3.36	3044.10	905.03	0.00
**PDGFA**	Platelet Derived Growth Factor Subunit A	-1.11	4.55	4.10	0.80
**PDGFB**	Platelet Derived Growth Factor Subunit B	1.80	1.12	2.02	0.28
**PDGFC**	Platelet Derived Growth Factor Subunit C	16.76	1.31	21.89	0.00
**PDGFD**	Platelet Derived Growth Factor Subunit D	3.67	0.90	3.30	0.00
**VEGFA**	Vascular Endothelial Growth Factor A	1.41	231.89	327.63	0.05
**VEGFB**	Vascular Endothelial Growth Factor B	-1.07	24.40	22.87	0.74
**VEGFC**	Vascular Endothelial Growth Factor C	3.27	1.08	3.53	0.00

9. Fortier, L. A., Barker, J. U., Strauss, E. J., McCarrel, T. M. & Cole, B. J. The role of growth factors in cartilage repair. in *Clinical Orthopaedics and Related Research* (2011). doi:10.1007/s11999-011-1857-3

Supplementary Table 3. Summary of overlapping genes between our RNAseq data and previous microarray data of healthy human chondrocytes treated by IL-1β [10]. The "-" in front of the fold change value represents downregulation of expression, and "+" represents upregulation of expression after IL-1β treatment.

	**Our RNAseq data**				**Microarray data [9]**			
**Gene symbol**	**Fold change**	**RPKM CTRL**	**RPKM IL-1b**	**FDR**	**Gene symbol**	**Fold change (low - high)**		**p value**
**BIRC3**	11.11	21.62	240.13	0.00	BIRC3	7.00	67.70	<0.001
**CAV1**	1.42	17.77	25.20	0.12	CAV1	4.20	39.80	<0.001
**CD74**	2.30	0.91	2.09	0.11	CD74	1.90	24.40	<0.001
**CD82**	2.09	60.98	127.25	0.00	CD82	2.00	12.30	<0.001
**CDKN1A**	12.34	8.78	108.33	0.00	CDKN1A	1.60	5.20	<0.001
**COL2A1**	-1.82	224154.50	123048.30	0.01	COL2A1	-2.50	0.00	<0.001
**CRKL**	1.15	2.04	2.34	0.74	CRKL	1.90	26.10	<0.005
**DAB2**	-1.24	69.72	56.34	0.29	DAB2	-2.00	-10.00	<0.005
**DYRK3**	1.84	4.16	7.66	0.00	DYRK3	1.90	26.20	<0.005
**FGF2**	3.30	4.92	16.25	0.00	FGF2	2.20	6.70	<0.001
**FOSL1**	3.53	1.03	3.64	0.00	FOSL1	2.60	6.00	<0.001
**FRZB**	-3.62	828.70	228.68	0.00	FRZB	-2.00	-10.00	<0.005
**HMGA1**	8.32	3.36	27.97	0.00	HMGA1	1.60	3.60	<0.001
**IFNGR2**	3.09	52.17	161.02	0.00	IFNGR2	5.20	44.60	<0.001
**IRF1**	14.88	0.16	2.38	0.00	IRF1	4.00	38.30	<0.001
**JUN**	1.10	106.23	116.76	0.81	JUN	3.00	13.80	<0.001
**KPNB1**	-1.19	641.98	537.44	0.31	KPNB1	-1.67	-3.33	<0.01
**LIF**	232.38	0.13	30.21	0.00	LIF	110.90	1420.20	<0.001
**MAPK9**	-1.16	11.53	9.91	0.56	MAPK9	-1.67	-3.33	<0.001
**MET**	2.60	1.14	2.96	0.02	MET	1.80	24.50	<0.01
**MMP13**	41.49	4.80	199.14	0.00	MMP13	4.30	15.50	<0.001
**NFKB1**	4.66	14.80	68.96	0.00	NFKB1	2.90	39.10	<0.005
**NFKB2**	7.11	16.63	118.32	0.00	NFKB2	3.10	18.50	<0.001
**NFKBIA**	11.60	23.51	272.61	0.00	NFKBIA	5.00	9.70	<0.001
**NTRK2**	-6.61	14.54	2.20	0.00	NTRK2	-5.00	0.00	<0.005
**PGF**	-1.09	7.80	7.13	0.71	PGF	4.60	43.00	<0.001
**RARG**	-1.76	248.25	141.16	0.00	RARG	-5.00	0.00	<0.001
**RND3**	1.47	10.46	15.40	0.09	RND3	3.60	12.00	<0.001
**SERPINE2**	2.81	197.01	554.38	0.00	SERPINE2	1.50	2.20	<0.001
**SGK1**	14.05	17.67	248.20	0.00	SGK1	6.60	54.30	<0.001
**SPARC**	-1.17	23049.56	19711.75	0.35	SPARC	-1.67	-5.00	<0.001
**TNFRSF1B**	15.93	1.63	25.97	0.00	TNFRSF1B	1.70	23.90	<0.01
**UPP1**	1.95	2.25	4.39	0.02	UPP1	2.60	32.60	<0.001
**VIM**	-1.40	1308.73	934.81	0.09	VIM	-1.67	-5.00	<0.001

10. Aigner, T. *et al.* Gene expression profiling of serum- and interleukin-1 beta-stimulated primary human adult articular chondrocytes - a molecular analysis based on chondrocytes isolated from one donor. *Cytokine* **31**, 227–240 (2005).

Supplementary Table 4. Summary of overlapping genes between our RNAseq data and previous microarray data of early osteoarthritic cartilage [11]. The "-" in front of the fold change value represents downregulation of expression, and "+" represents upregulation of expression after IL-1β treatment.

	Our RNAseq data				Microarray data [10]			
**Gene symbol**	**Fold change**	**RPKM CTRL**	**RPKM IL-1b**	**FDR**	**Gene symbol**	**Fold change**	**q value**	**p value**
**COPS6**	-1.18	62.73	53.20	0.32	COPS6	-1.75	0.00	0.00
**FN1**	6.61	3869.11	25578.18	0.00	FN1	1.58	0.00	0.00
**FOS**	2.07	12.08	24.95	0.02	FOS	1.88	0.00	0.00
**FUS**	-1.49	244.17	164.03	0.00	FUS	-1.82	0.02	0.00
**KDELR2**	-1.70	361.34	212.91	0.00	KDELR2	-1.82	0.03	0.00
**PLRG1**	1.04	22.71	23.67	0.83	PLRG1	1.92	0.04	0.00
**RAB1A**	-1.21	149.37	123.60	0.24	RAB1A	-1.75	0.03	0.00
**RABAC1**	-1.18	134.28	113.58	0.40	RABAC1	-1.54	0.00	0.00
**TRADD**	-1.16	8.76	7.52	0.49	TRADD	-1.72	0.03	0.00
**TSC22D3**	-1.14	48.63	42.83	0.58	TSC22D3	-1.82	0.04	0.00

11. Aigner, T. *et al*. Large-scale gene expression profiling reveals major pathogenetic pathways of cartilage degeneration in osteoarthritis. *Arthritis Rheum.* **54**, 3533–3544 (2006).

Supplementary Table 5. Summary of reference genes that have been commonly used in cartilage studies. The most widely accepted housekeeping genes in chondrocyte and cartilage studies include the glyceraldehyde-3-phosphate dehydrogenase (GAPDH), cyclophilin (CYP), ribosomal protein L4 (RPL4), hypoxanthine phosphoribosyl transferase (HPRT1), β-2-microglobulin (B2M), actin β (ACTB), and tyrosine 3-Monooxygenase (YWHAZ). In our RNAseq data, the basal mRNA levels of ACTB, RPL4 and GAPDH in bovine chondrocytes were substantial (RMPK value: 1137.52, 692.26, and 485.27), while RMPK of the other genes were below 100. After the treatment of IL-1β, ACTB and GAPDH showed minor changes (-1.19 folds for ACTB; 1.08 folds for GAPDH; FDR > 0.05), which indicates that these two genes are suitable reference genes for bovine cartilage.

Gene symbol	Gene expression				
	Down (-) /Up(+)	Fold change (IL-1β/CTRL)	RPKM (CTRL)	RPKM (IL-1β)	FDR
**YWHAZ**	-	-1.07	3.72	3.47	0.85
**HADHB**	-	-1.00	43.04	42.93	0.99
**HPRT1**	-	-1.23	67.28	56.26	0.35
**PPIA**	-	-1.23	73.68	59.00	0.19
**SDHA**	-	-1.05	79.56	75.88	0.80
**GAPDH**	+	1.10	485.27	523.00	0.66
**RPL4**	+	1.44	692.26	988.90	0.02
**ACTB**	-	-1.20	1137.52	953.64	0.27

Supplementary Figure 1. Mechanical properties of cartilage explants after 2-day IL-1β treatment determined by unconfined compression test, including equilibrium Young's modulus, dynamic modulus, and permeability.

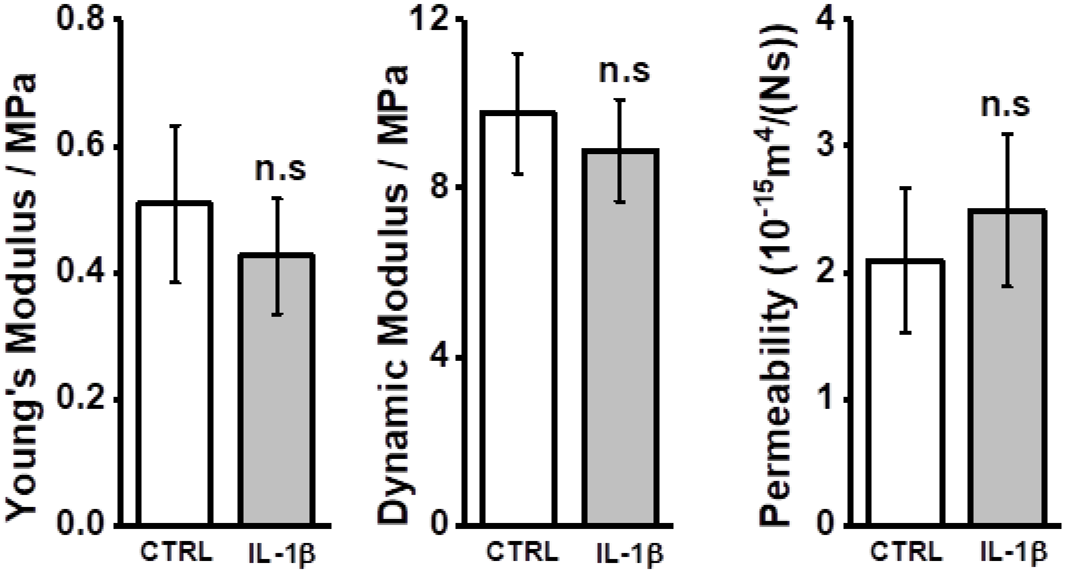

Supplementary Figure 2. Visualization of pathways regulated by IL-1β using our RNA sequencing data. (a) cell cycle, (b) cellular senescence, (c) autophagy, (d) p53 signaling pathway, (e) regulation of actin cytoskeleton, (f) chemokine signaling pathway, (g) JAK-STAT signaling pathway, and (h) inflammatory mediator regulation of TRP channels signaling pathway.

The pathway flowcharts were generated by KEGG database [12]. The KEGG pathway is a molecular interaction/reaction network diagram, which is drawn according to experimental evidence from previous publications. The color profile of each node represents the upregulation/downregulation detected in our RNAseq data ($\log_2(|fold\ change|)$). Blue color denotes downregulation and red color denotes upregulation in the IL-1β-treated cartilage compared to the control group. The nodes without color are genes that were not detected in our RNAseq data set.

a. Cell cycle progression is accomplished through a reproducible sequence of events, DNA replication (S phase) and mitosis (M phase) separated temporally by gaps known as G1 and G2 phases. The KEGG pathway has 105 nodes with 101 mapped to our data set.

In healthy cartilage, chondrocytes remain in a resting state and refrain from proliferation or terminal differentiation. In a diseased state, however, some articular chondrocytes enter into abnormal cell cycle progression, which is characterized by a cascade of proliferation and hypertrophic differentiation, with subsequent cell apoptosis and mineralization of the diseased cartilage. This aberrant cell cycle transition is often associated with elevated expression and release of proteases, such as MMP-13 and ADAMTS-5, leading to cartilage degeneration and OA development [13].

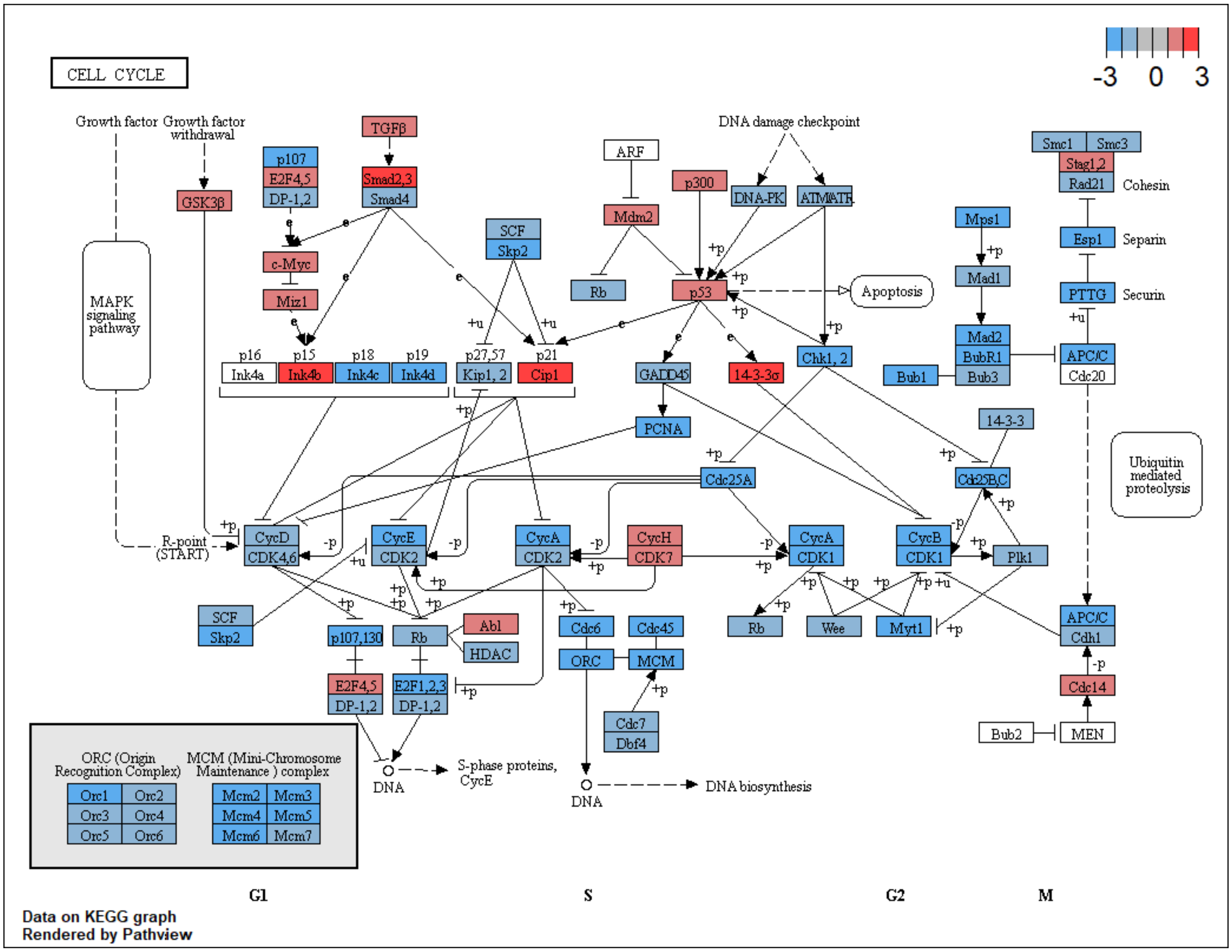

b. Cellular senescence is a state of irreversible cellular arrest. It is characterized by enlarged morphology, senescence-associated beta-galactosidase (SA-b-gal) activity, as well as the secretion of inflammatory cytokines, growth factors and matrix MMPs. These features are part of the senescence-associated secretory phenotype (SASP). This KEGG pathway has 97 nodes with 89 mapped to our data set.

Cellular senescence plays a significant role in the pathology of OA. Osteoarthritic chondrocytes often exhibit a variety of senescent-associated phenotypes, leading to disturbed balance between ECM synthesis and degradation. Aging is believed to be one of the major risk factors inducing chondrocytes senescence of OA cartilage. During aging, chondrocytes exhibit many features of senescence, including growth arrest as well as decreased proliferation capacity and ECM synthesis. Another important factor that induces senescence is environmental stress, *i.e.*, oxidative stress, cytokines inflammatory stimulation, and mechanical stress. The stress-induced senescence can lead to chondrocyte clustering, proliferation, and increased release of MMPs enzymes [14].

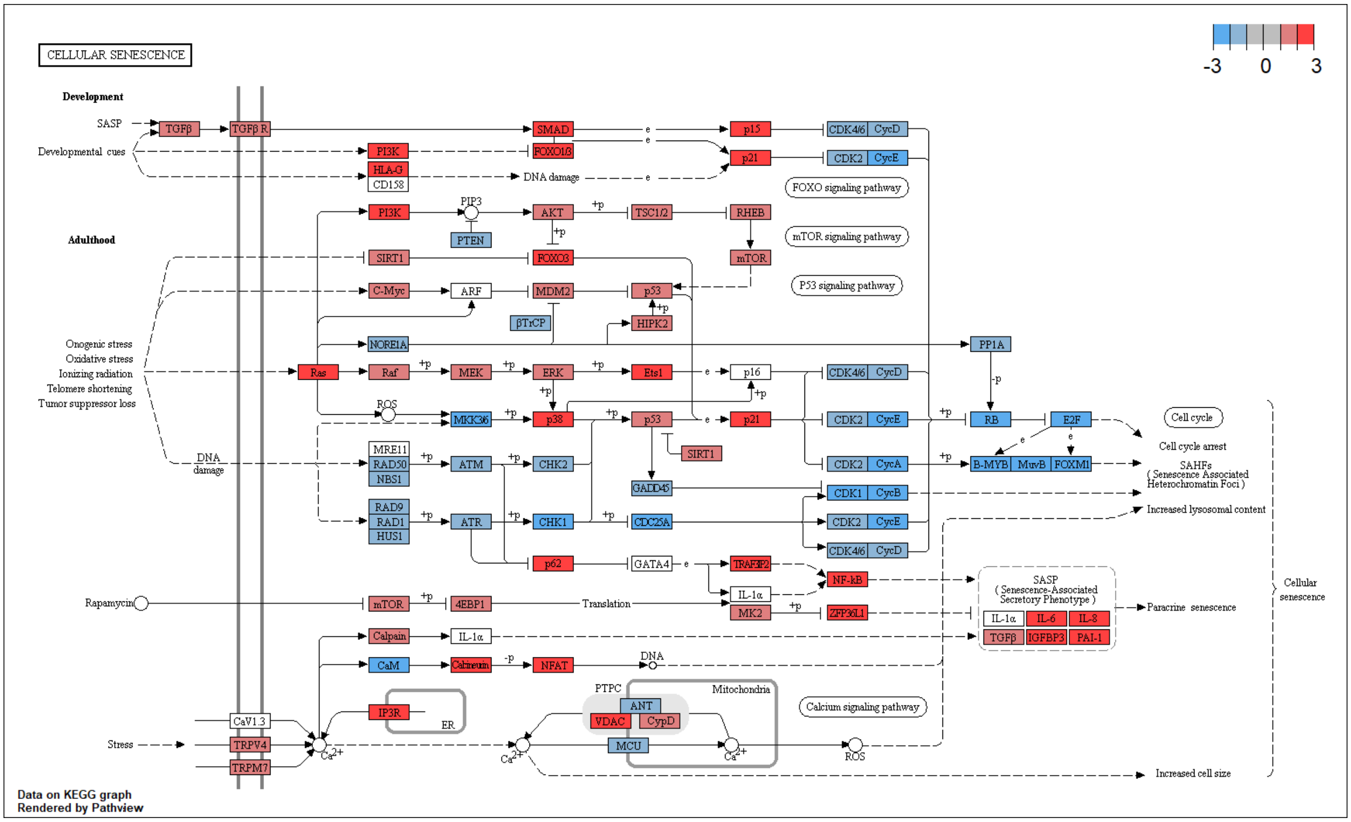

c. Autophagy is a cellular catabolic activity related to protein degradation, organelle turnover, and non-selective breakdown of cytoplasmic components. Constitutive level of autophagy plays a key role in cellular homeostasis and maintains the quality of essential cellular components. The KEGG pathway has 104 nodes with 88 mapped to our data set.

Autophagy is a protective mechanism in normal cartilage; however, compromised autophagy activity is associated with OA development. In normal cartilage, chondrocyte autophagy is constitutively active and apparently protective process for the maintenance of the homeostatic state. By contrast, human OA as well as aging-related and surgically-induced mice OA cartilage present a significant reduction and loss of expression of *Atg* genes (ULK1, Beclin1 and LC3), which are major regulators of the autophagy pathway [15]. These results suggest that compromised autophagy represents an important mechanism in the development of OA.

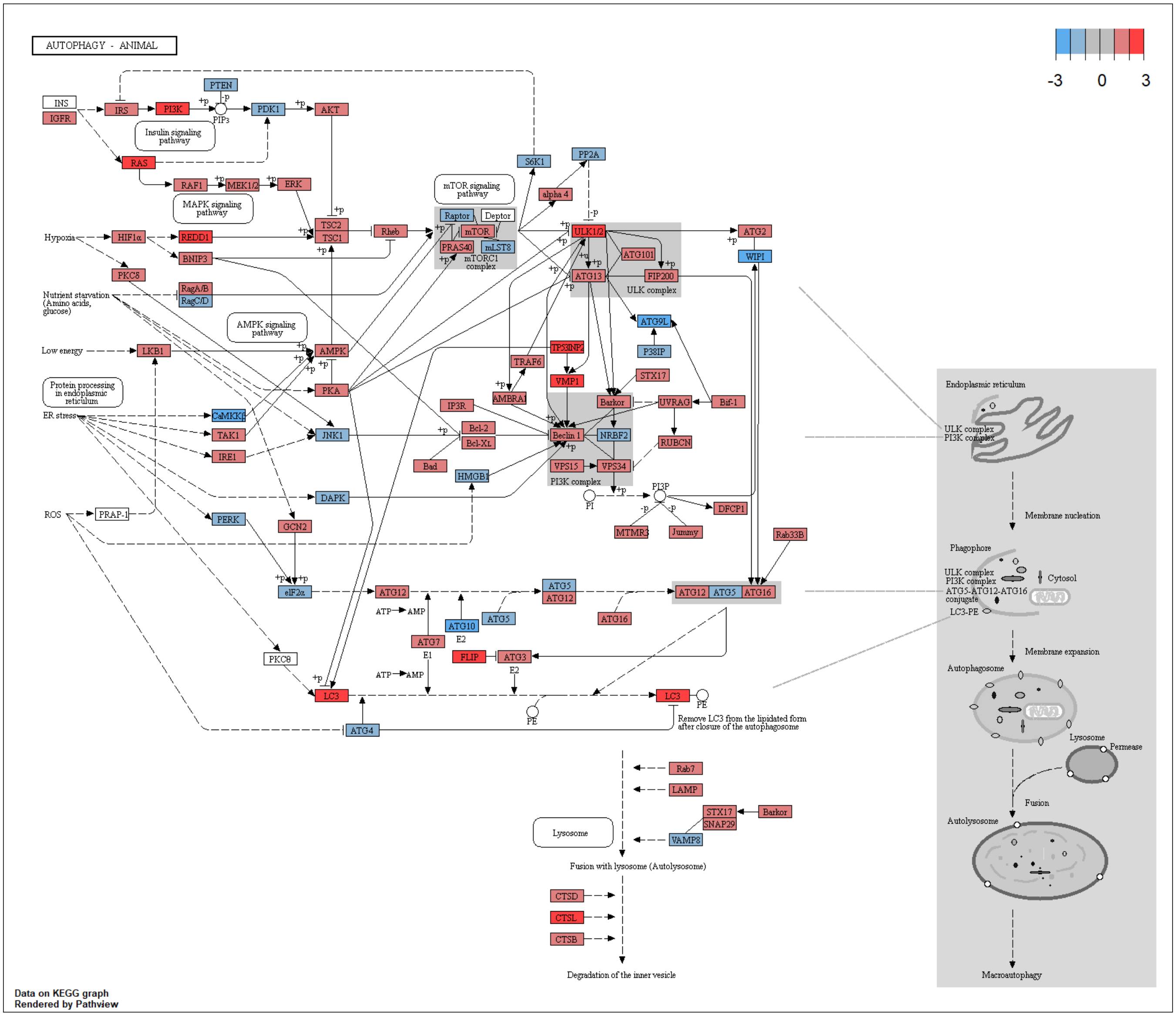

d. p53 activation is induced by a few stress signals and related to three major events, cell cycle arrest, cellular senescence, and apoptosis. The KEGG pathway has 61 nodes with 52 mapped to our data set.

P53 is also known as p53 tumor suppressor protein, which can regulate the cell cycle and hence function as tumor suppression. During OA progression, the apoptosis of human chondrocytes is accompanied with the expression of p53 [16]. Another study using rabbit articular cartilage indicates that nitric oxide, an important inducer of apoptosis, can induce chondrocyte apoptosis with the activation of p53 and caspase3 [17]. In another *in vitro* study, IL-1β induces chondrocyte apoptosis with the accumulation of p53 [18].

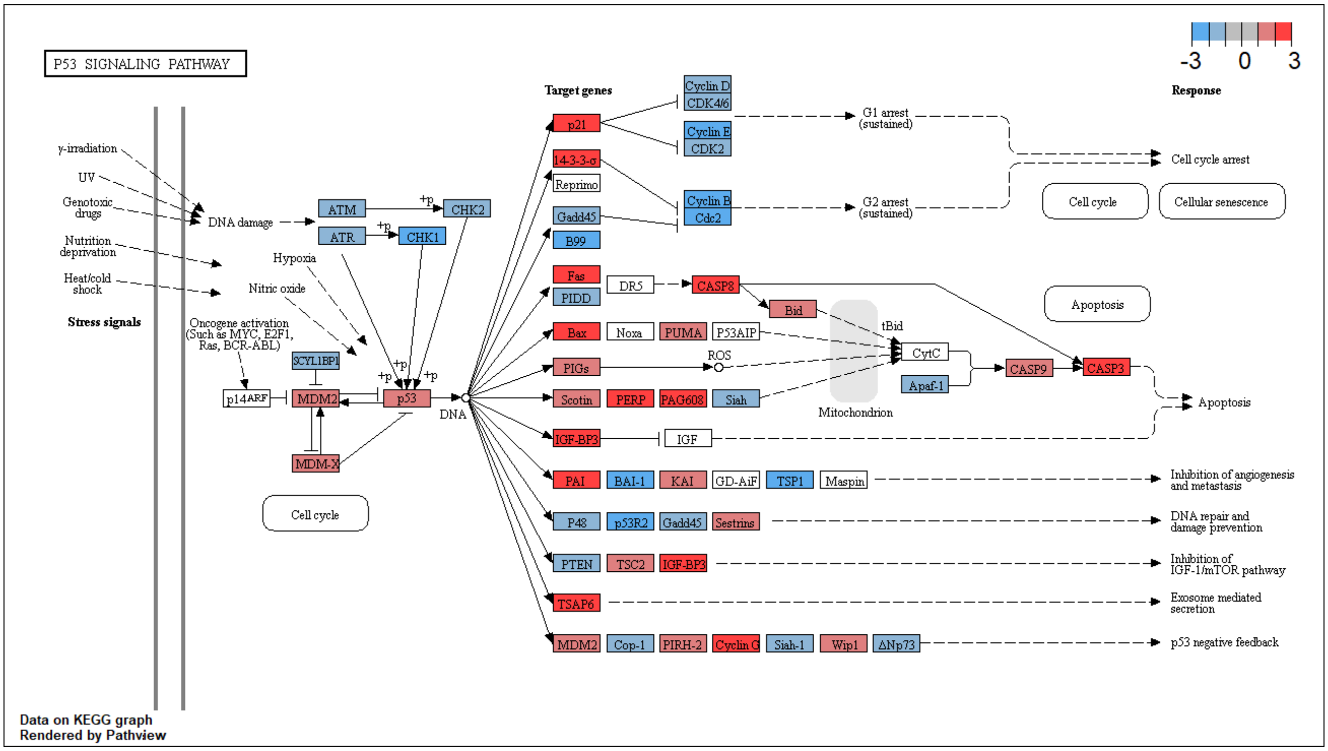

e. Dynamic remolding of the actin cytoskeleton regulates many cellular activities. Dynamic transition exists between two forms of actin, monomeric (G-actin) and filamentous (F-actin). The KEGG pathway has 71 nodes with 68 mapped to our data set.

Cell morphology and the associated cytoskeleton organization can influence chondrocyte metabolism. Chondrocyte cytoskeleton is subject to reorganization under biomechanical stimuli or OA progression. Cytokines and growth factors can manipulate the dynamics of cytoskeleton, which can further modulate the biosynthetic activities of chondrocytes. One of the elucidated mechanisms of cytokine-induced actin reorganization has been shown to involve activation of PKC (protein kinase C) in a Rho-dependent manner [19]. In brief, Rho GTPases, such as Rac1, RhoA and Cdc42, can be activated and regulate a variety of effectors, including PKC and some actin-binding proteins. These direct or indirect interactions can affect the local assembly or disassembly of filamentous (F)-actin.

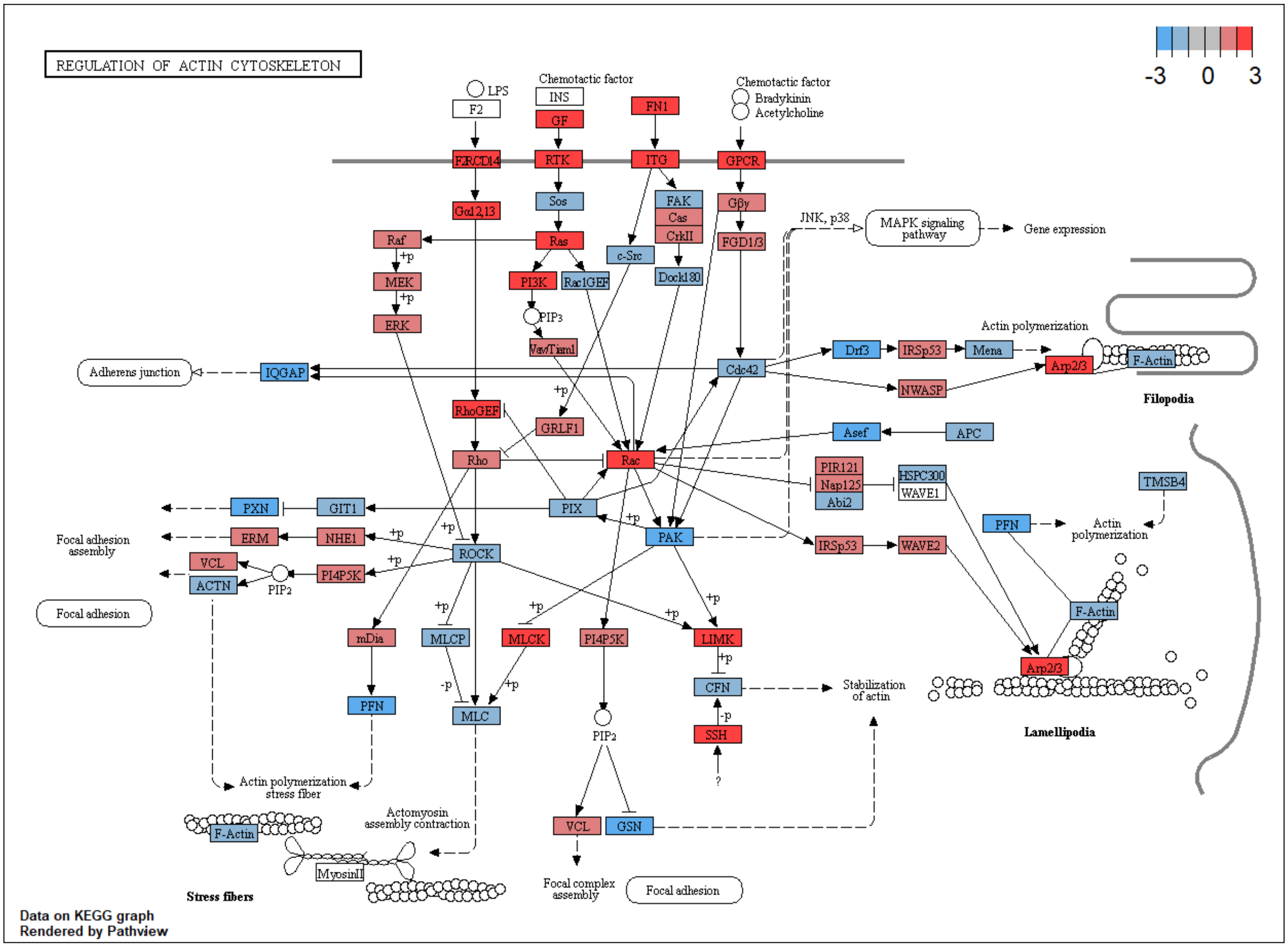

f. Chemokines are a family of small cytokines or signaling proteins secreted by cells. They can induce directed chemotaxis in nearby responsive cells; they are chemotactic cytokines. The chemokine signal is transduced by chemokine receptors (G-protein coupled receptors). After receptor activation, the alpha- and beta-gamma-subunits of G protein dissociate to activate diverse downstream pathways and result in cellular polarization and actin reorganization.

In cartilage, chondrocytes can express chemokines and chemokine receptors, which play important roles in activating catabolic pathways and chondrocyte hypertrophy. The level of chemokines is highly elevated after joint injuries or at the early stage of OA. Chemokines can synergize with cytokines and promote chondrocyte catabolic activities, leading to cartilage ECM degradation. The degraded cartilage matrix proteins or fragments, in turn, can activate certain receptors and stimulate the production of matrix-degrading proteases, inflammatory cytokines, and chemokines, forming a self-boost amplification process [20].

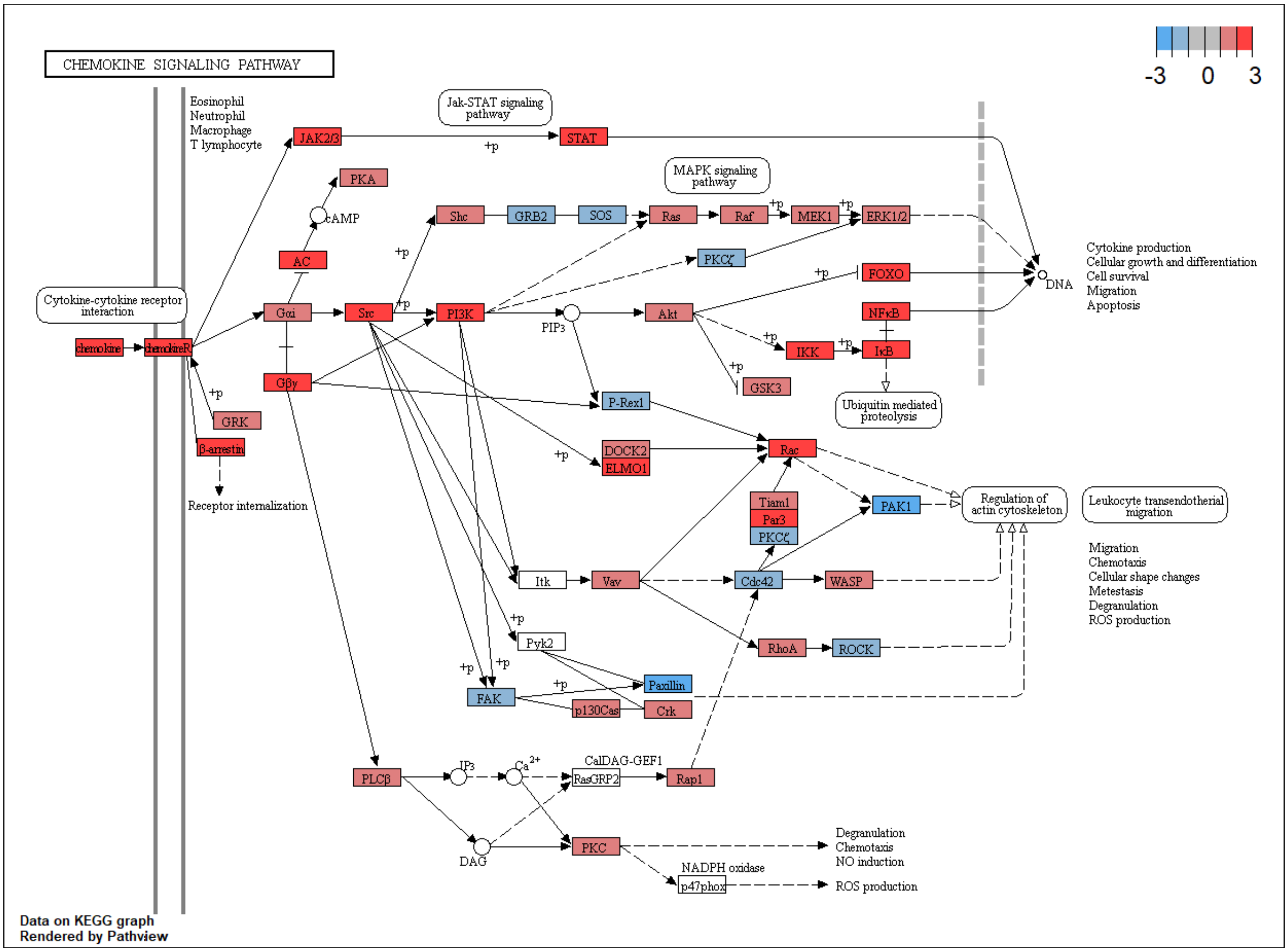

g. JAK/STAT (Janus kinase/signal transducers and activators of transcription) pathway is a principal signaling mechanism involving a wide array of cytokines and growth factors. Once activated, STATs and JAK can modulate the expression of target genes and mediate the recruitment of molecules such as the MAP kinases and PI3 kinase. These molecules process downstream signals via the Ras-Raf-MAP kinase and PI3 kinase pathways, which can result in the activation of additional transcription factors. This KEGG pathway has 35 nodes with 35 mapped to our data set.

In chondrocytes, IL-6-induced downregulation of type II collagen, aggrecan and link proteins is via JAK/STAT pathway [21]. In addition, JAK-STAT signaling pathway also plays a critical role in regulating the pain in inflammatory arthritis. Activation of JAK/STAT by pro-inflammatory cytokines is considered to be of major importance in driving chronic inflammation in joint tissues [22].

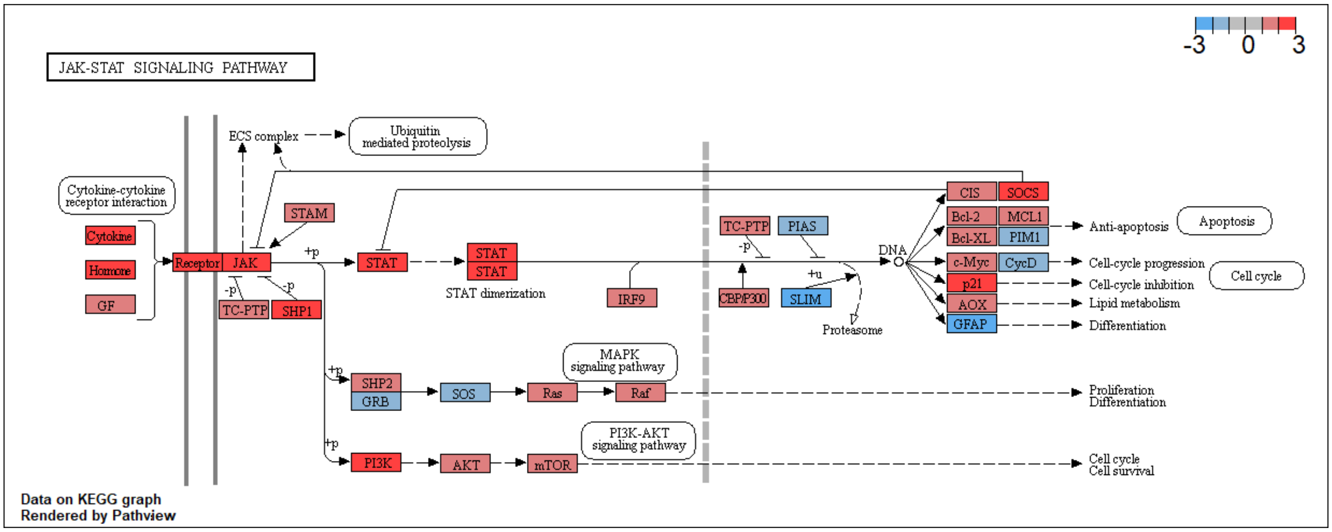

h. Toll-like receptors (TLRs) were initially discovered in innate immune cells under bacteria stimulation. Activated TLRs provoke rapid activation of innate immunity by inducing production of proinflammatory cytokines and upregulation of co-stimulatory molecules. This KEGG pathway has 79 nodes with 68 mapped to our data set.

TLRs are expressed in chondrocytes and synoviocytes, which can be activated by inflammatory stimuli. The stressed cells can synthesize and secrete molecules called damage-associated molecular patterns (DAMPs), which act as ligands for TLRs inducing inflammatory and catabolic responses in articular cartilage. In chondrocytes, the S100 calcium-binding proteins (S100A4, A8, A9, and A11) have been identified as DAMPs. They can activate TLR2 and TLR4, leading to increased expression of catabolic genes, such as MMP-3, MMP-13 and NOS2, through upregulating cytokine and chemokine expression [20].

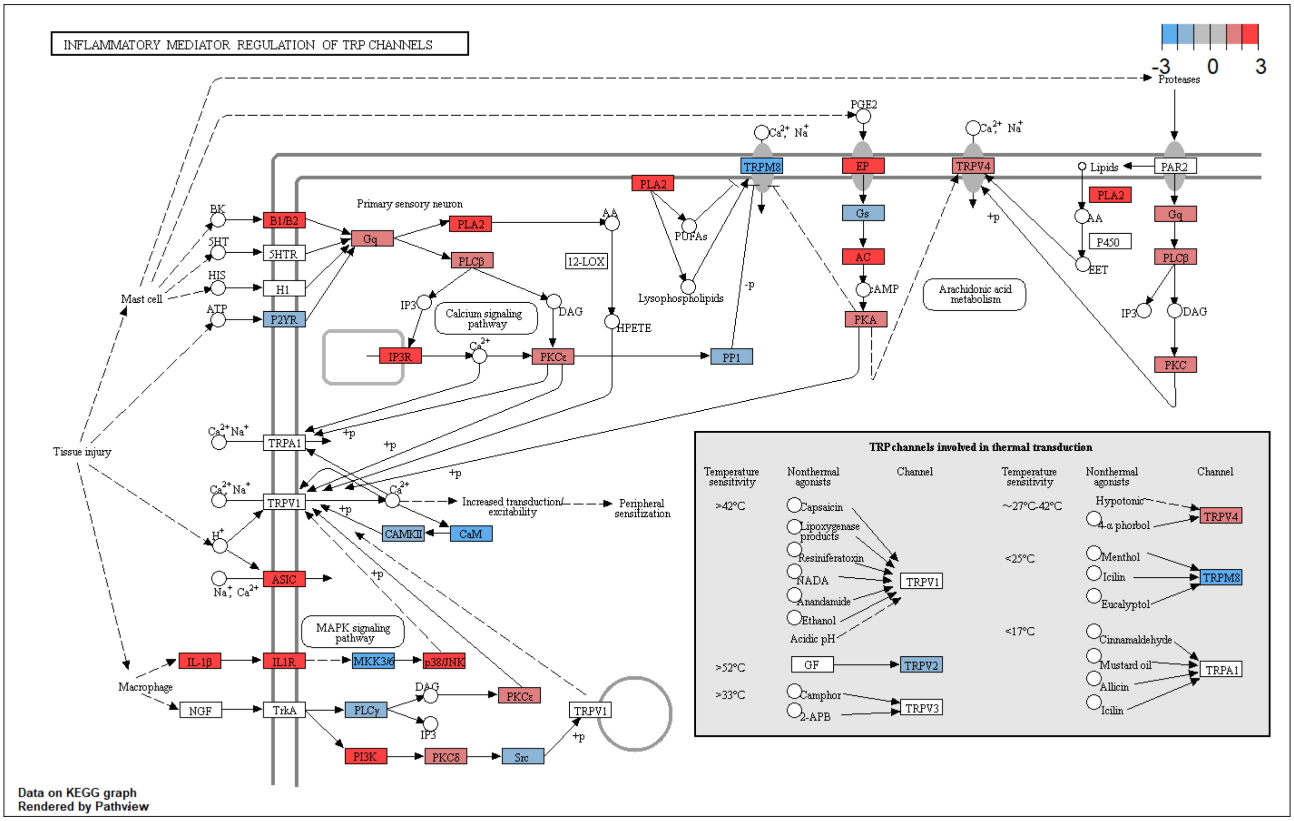

12. Kanehisa, M., Furumichi, M., Tanabe, M., Sato, Y. & Morishima, K. KEGG: New perspectives on genomes, pathways, diseases and drugs. *Nucleic Acids Res.* (2017). doi:10.1093/nar/gkw1092

13. Dreier, R. Hypertrophic differentiation of chondrocytes in osteoarthritis: The developmental aspect of degenerative joint disorders. *Arthritis Research and Therapy* **12,** (2010).

14. Vinatier, C., Domínguez, E., Guicheux, J. & Caramés, B. Role of the Inflammation-Autophagy-Senescence Integrative Network in Osteoarthritis. *Front. Physiol.* **9,** 706 (2018).

15. Caramés, B., Taniguchi, N., Otsuki, S., Blanco, F. J. & Lotz, M. Autophagy is a protective mechanism in normal cartilage, and its aging-related loss is linked with cell death and osteoarthritis. *Arthritis Rheum.* (2010). doi:10.1002/art.27305

16. Hashimoto, S. *et al.* Role of p53 in human chondrocyte apoptosis in response to shear strain. *Arthritis*

Rheum. (2009). doi:10.1002/art.24706

17. Sung, W. L. *et al.* Cilostazol protects rat chondrocytes against nitric oxide-induced apoptosis in vitro and prevents cartilage destruction in a rat model of osteoarthritis. *Arthritis Rheum.* (2008). doi:10.1002/art.23220

18. Csaki, C., Keshishzadeh, N., Fischer, K. & Shakibaei, M. Regulation of inflammation signalling by resveratrol in human chondrocytes in vitro. *Biochem. Pharmacol.* (2008). doi:10.1016/j.bcp.2007.09.014

19. Blain, E. J. Involvement of the cytoskeletal elements in articular cartilage homeostasis and pathology. *International Journal of Experimental Pathology* (2009). doi:10.1111/j.1365-2613.2008.00625.x

20. Goldring, M. B. Chondrogenesis, chondrocyte differentiation, and articular cartilage metabolism in health and osteoarthritis. *Ther. Adv. Musculoskelet. Dis.* (2012). doi:10.1177/1759720X12448454

21. Legendre, F., Dudhia, J., Pujol, J.-P. & Bogdanowicz, P. JAK/STAT but not ERK1/ERK2 pathway mediates interleukin (IL)-6/soluble IL-6R down-regulation of Type II collagen, aggrecan core, and link protein transcription in articular chondrocytes. Association with a down-regulation of SOX9 expression. *J. Biol. Chem.* **278,** 2903–12 (2003).

22. Malemud, C. J. The role of the JAK/STAT signal pathway in rheumatoid arthritis. *Ther. Adv. Musculoskelet. Dis.* **10,** 117–127 (2018).
